# *Mustn1* ablation in skeletal muscle results in functional alterations

**DOI:** 10.1096/fba.2023-00082

**Published:** 2023-11-15

**Authors:** Charles J. Kim, Chanpreet Singh, Marina Kaczmarek, Madison O'Donnell, Christine Lee, Kevin DiMagno, Melody W. Young, William Letsou, Raddy L. Ramos, Michael C. Granatosky, Michael Hadjiargyrou

**Affiliations:** ^1^ College of Osteopathic Medicine New York Institute of Technology Old Westbury New York USA; ^2^ Department of Biological and Chemical Sciences New York Institute of Technology Old Westbury New York USA; ^3^ Department of Anatomy, College of Osteopathic Medicine New York Institute of Technology Old Westbury New York USA; ^4^ Department of Biomedical Sciences, College of Osteopathic Medicine New York Institute of Technology Old Westbury New York USA; ^5^ Center for Biomedical Innovation New York Institute of Technology Old Westbury New York USA

**Keywords:** gait alterations, grip strength, isometric contraction tests, knockout mice, muscle fiber type, Mustn1, Pax7, skeletal muscle

## Abstract

*Mustn1*, a gene expressed exclusively in the musculoskeletal system, was shown in previous in vitro studies to be a key regulator of myogenic differentiation and myofusion. Other studies also showed *Mustn1* expression associated with skeletal muscle development and hypertrophy. However, its specific role in skeletal muscle function remains unclear. This study sought to investigate the effects of *Mustn1* in a conditional knockout (KO) mouse model in Pax7 positive skeletal muscle satellite cells. Specifically, we investigated the potential effects of *Mustn1* on myogenic gene expression, grip strength, alterations in gait, ex vivo investigations of isolated skeletal muscle isometric contractions, and potential changes in the composition of muscle fiber types. Results indicate that *Mustn1* KO mice did not present any substantial phenotypic changes or significant variations in genes related to myogenic differentiation and fusion. However, an approximately 10% decrease in overall grip strength was observed in the 2‐month‐old KO mice in comparison to the control wild type (WT), but this decrease was not significant when normalized by weight. KO mice also generated approximately 8% higher vertical force than WT at 4 months in the hindlimb. Ex vivo experiments revealed decreases in about 20 to 50% in skeletal muscle contractions and about 10%–20% fatigue in soleus of both 2‐ and 4‐month‐old KO mice, respectively. Lastly, immunofluorescent analyses showed a persistent increase of Type IIb fibers up to 15‐fold in the KO mice while Type I fibers decreased about 20% and 30% at both 2 and 4 months, respectively. These findings suggest a potential adaptive or compensatory mechanism following *Mustn1* loss, as well as hinting at an association between *Mustn1* and muscle fiber typing. Collectively, *Mustn1*'s complex roles in skeletal muscle physiology requires further research, particularly in terms of understanding the potential role of *Mustn1* in muscle repair and regeneration, as well as with influence of exercise. Collectively, these will offer valuable insights into *Mustn1*'s key biological functions and regulatory pathways.

## Graphical abstract

Mustn1 and skeletal muscle.

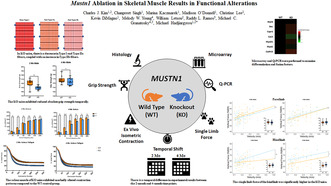

## 1 INTRODUCTION

Locomotion, a critical function of animal life, relies heavily on the integrity of the skeletal muscle system, which constitutes roughly 40% of body mass. The coordination of skeletal muscles allow for the precise movements needed for grip and gait, and is regulated by complex genetic networks.^1^, ^2^, ^3^ Dysregulation of normal genetic networks may result in weakness or pathologies linked to skeletal muscle diseases.^4^ Functional skeletal muscle is primarily formed by multinucleated myofibers, which originate from the differentiation and fusion of myocytes (myoblasts) during embryonic development. Postnatal skeletal muscle growth and repair partially rely on a small population of myogenic precursor satellite cells. In many ways, postnatal myogenesis mirrors embryogenesis, as satellite cells undergo activation, migration, proliferation, differentiation, and fusion, ultimately leading to myofibers and repairing or hypertrophy of mature skeletal muscle.^5^

Given the vital role of functional skeletal muscles in maintaining physical and metabolic capacities, it is crucial to understand how dysregulation of myogenesis may lead to skeletal muscle function impairment. Therefore, investigating the fundamental molecular processes of myogenesis, involving potential regulatory genes such as *Mustn1*,^6^ is essential for devising future comprehensive strategies to prevent and treat skeletal muscle wasting (e.g., diabetes, sarcopenia, dystrophies), and injuries arising from direct (e.g., lacerations, contusions, and strains) and indirect (e.g., ischemia and neurological dysfunction) causes.^7^, ^8^

The primary objective of this project was to elucidate the functional contribution of *Mustn1* as a positive regulator of skeletal muscle development and function. *Mustn1* was initially identified as one of the upregulated genes during fracture repair^9^ and has since been recognized as a pan‐musculoskeletal gene expressed by cells that form bone, cartilage, tendon, and skeletal muscle. Currently, *Mustn1* is the only known pan‐musculoskeletal cell maker.^6^

Prior investigations dealing with cloning and expression,^9^ promoter characterization,^10^ and functional perturbation of *Mustn1* showed that it is expressed exclusively in the musculoskeletal system, especially in adult skeletal muscle and tendon (and to a lesser degree in bone and cartilage)^9^ as well as in developing skeletal muscle in various species^11^, ^12^, ^13^, ^14^, ^15^, ^16^, ^17^, ^18^, ^19^, ^20^ and during myogenic differentiation of C2C12 cells.^21^ Further, *Mustn1* expression during skeletal muscle regeneration was also demonstrated,^22^ while other laboratories reported data showing that *Mustn1* expression is upregulated during muscle exercise and hypertrophy.^23^, ^24^, ^25^, ^26^ Functional perturbation via silencing impaired differentiation and myotube formation as well as downregulating the expression of myogenic differentiation and fusion markers.^21^ Further, its downregulation resulted in in vivo developmental malformations in body axis and tail of *Xenopus*.^27^ Hu et al.^28^ also showed that *Mustn1* is an important molecular regulator for muscle growth and development, particularly in proliferation and differentiation of skeletal muscle satellite cells in chickens. Finally, we recently also demonstrated that *Mustn1* ablation in skeletal muscle results in increased glucose tolerance concomitant with upregulated GLUT expression.^29^

Although the precise molecular function of *Mustn1* remains unclear, its expression pattern and functional perturbation in vitro and in vivo strongly suggests that it may positively regulate myogenic differentiation and fusion, contributing to fiber formation and, ultimately, skeletal muscle development. As such, we assessed the functional role of *Mustn1* by generating a conditional KO mouse model where *Mustn1* was ablated in Pax7‐positive skeletal muscle satellite cells.

## 2 MATERIALS AND METHODS

All animal care and experimental procedures were conducted in strict adherence to the guidelines established by the Institutional Animal Care and Use Committee (IACUC) and their approved protocols. The generation of *Mustn1* conditional KO mice, specifically targeting skeletal muscle, along with the methodological details and validation of the model, has been previously reported by our laboratory.^29^ The study described herein focused exclusively on male mice, given the absence of observable differences in female mice across all experimental conditions, as previously reported.^29^ Moreover, this study involved a comparative analysis between two groups of male mice, WT and KO, at two crucial developmental stages: 2 and 4 months of age. These time points were strategically selected to assess the impact of *Mustn1* during its peak expression period, as they correspond to 1 month before and 1 month after the third month, which is recognized as the peak of *Mustn1* expression.^21^ Mice were housed in groups of 4–5 per cage in a central specific pathogen‐free facility and kept in 12:12 h light and dark cycles with ad libitum access to standard chow and water.

### 2.1 Microarray and bioinformatics

We previously described the use utilization of microarray to determine differential gene expression between the KO and WT mice.^29^ Specifically, RNA was isolated from the right gastrocnemius muscles that were excised from hindlimbs of mice (*n* = 3/group). Each extracted muscle was immediately immersed in TRIzol™ Reagent (Invitrogen) at a ratio of 1 mL reagent for every 50–100 mg of tissue, adhering to the manufacturer's instructions. To prevent sample degradation, tissue samples were immediately homogenized using a motorized tissue homogenizer maintained at 4°C. RNA extraction was executed as outlined in the manufacturer's protocol (ThermoFisher, 2020). Post‐extraction, the RNA samples underwent purification with the RNeasy Plus Mini Kit (Qiagen), following the manufacturer's guidelines (Qiagen). RNA integrity was subsequently verified through gel electrophoresis, and their concentrations ascertained with a NanoDrop 2000c spectrophotometer. For microarray analysis, individual RNA samples (*n* = 3) from the same genotype were pooled to form the final RNA sample. The GeneChip™ WT PLUS Reagent Kit (Applied Biosystems) was used, according to manufacturer's protocol, to prepare 150 ng of the pooled total RNA for whole transcriptome expression analysis. Following hybridization washing and staining (according to manufacturer's protocol), the microarrays were then scanned in a GeneChip Scanner 3000 G7 controlled by GeneChip Command Console v 4.3.3.1616 software and the data was analyzed using Applied BioSystems Transcriptome Analysis Console software v4.0.2.15.

Differential gene expression was analyzed using R version 4.2.1 and visualized in ggplot2.^30^ Genes were ordered by expression in the WT condition, and a fold change of 2 (up or downregulated) in the KO condition was taken as the cutoff for differential expression.

### 2.2 Quantitative real‐time polymerase chain reaction

Under sterile conditions, the right gastrocnemius muscle was excised from the hindlimb of each mouse (*n* = 4/group) at the terminal experiments. The isolated muscle samples were immediately submerged in TRIzol™ Reagent (Invitrogen); we used 1 mL per every 50–100 mg of tissue sample, as per manufacturer's protocol. Samples were immediately homogenized using a motorized tissue homogenizer at 4°C in order minimize sample degradation. Subsequent RNA extraction was performed following the manufacturer's standard protocol and guidelines (ThermoFisher, 2020). Following RNA extraction, samples were purified using the RNeasy Plus Mini Kit (Qiagen), per protocol provided by the manufacturer (Qiagen). RNA quality was determined by gel electrophoresis and concentration were measured using a NanoDrop 2000c spectrophotometer. cDNA was synthesized from 1 μg of purified RNA samples using ReadyScript™ cDNA Synthesis Mix (Sigma‐Aldrich) following manufacturer's protocol. Yield and purity were again assessed using a Nanodrop. Quantitative real‐time polymerase chain reaction (Q‐PCR) was carried out using LightCycler 480 SYBR Green I Master rt‐PCR kit (Roche) and the LightCycler system following a standard protocol provided by the manufacturer. Genes of interest and their primers are listed in Table 1.

**TABLE 1:** List of gene primer sequences used for Q‐PCR.

Target gene	Accession#	Primer sequence	Amplicon size (bp)
*18S*	NR 003278.3	Forward: CGCGGTTCTATTTTGTTGGT	219
		Reverse: AGTCGGCATCGTTTATGGTC	
*MyoD*	NM 010866.2	Forward: GCCTGAGCAAAGTGAATGAG	184
		Reverse: GGTCCAGGTGCGTAGAAGG	
*MyoG*	BC 068019.1	Forward: GGAAGTCTGTGTCGGTGGAC	150
		Reverse: CGCTGCGCAGGATCTCCAC	
*Myh4*	NM 010855.3	Forward: CAAGTCATCGGTGTTTGTGG	175
		Reverse: GGCCATGTCCTCAATCTTGT	
*Des*	BC 031760.1	Forward: GCGGCTAAGAACATCTCTGA	116
		Reverse: TCCATCATCTCCTGCTTGG	
*Capn1*	BC 050276.1	Forward: GGTGAAGTGGAGTGGAAAGG	226
		Reverse: TGCCCTCGTAAAATGTGGTA	
*Cav3*	NM 007617.3	Forward: ACGGTGTATGGAAGGTGAGC	203
		Reverse: TGAGTAGATGTGGCTGATGC	
*Cadh15*	BC 157978.1	Forward: CCCAACTAAGGGGCTCTCTC	150
		Reverse: ATTCTCCCACCACTCCTGACT	

### 2.3 Grip strength test

The grip strength test was conducted with 2‐ and 4‐month‐old KO and WT male mice (*n* = 10/group) using a modified forelimb grip strength test as described by Takeshita et al.,^31^ which has been demonstrated to exhibit greater sensitivity and reliability compared to conventional tests. A Grip Strength Meter (Item #761066; Harvard Apparatus, Holliston, MA) was employed to assess grip strength as the mouse grasped a vertically fixed bar. An experimenter steadily pulled the mouse's tail downward until the forepaws released the bar. The Grip Strength Meter recorded the peak force exerted by the mouse in grams (g).

Any trials in which the mouse utilized only one forepaw, any hindlimbs, bit the grip bar, turned during the pull, or released the bar without resistance were excluded from the analysis. The test consisted of six consecutive measurements per day, with 1‐min intervals between each trial. The order of testing was randomized daily, the experimenter was blinded to the genotype of the mice, and the same experimenter conducted all grip strength assessments to reduce variability. All test sessions took place during the afternoon hours of the light cycle (10 AM to 4 PM) in the animals' housing environment.

### 2.4 Single limb force analysis

Single limb force data were obtained from both WT and KO male mice (*n* = 5/group). Data collection adhered to protocols approved by the Institutional Animal Care and Use Committee. All animals, aged 2 or 4 months, exhibited no visible pathologies or gait abnormalities. Experimental procedures were adapted from arboreal gait studies in primates,^32^, ^33^, ^34^, ^35^, ^36^ small mammals,^37^, ^38^ and squamates^39^ and birds.^40^ Mice walked across a wooden runway instrumented with a small load force plate (model HE6X6; Advanced Mechanical Technology, Inc., Watertown, MA) calibrated with known‐mass weights prior to data collection. The instrumented portion were custom three‐dimensional printed platforms measuring 19 mm × 27 mm (scaled for the size a mouse fore‐/hindpaw). The instrumented sections were flush mounted and flanked by small gaps separating them from the non‐instrumented sections.

All trials commenced with HE6X6 force plate zeroed to eliminate drift or offsets. Mice were acclimated to the experimental setup to ensure normalcy when moving across the runway. Minimal motivation, such as a light tail tap or gentle blowing, was typically sufficient to initiate animal movement. Only trials in which the animal moved in a straight, continuous paths with no observable acceleration or deceleration were deemed successful trials. Among these, only trials with clear footfalls (i.e., single limb contacts with the instrumented platform) were retained for subsequent processing and analysis. The goal was to collect approximately 30 footfalls (15 forelimb and 15 hindlimb) per mouse on each substrate.

All trials were captured using high‐speed videography (XC‐2; Xcitex Inc., Woburn, MA) from lateral‐view at 125 Hz. Forces (sampled at 1250 Hz) and video data were synchronized using ProCapture. A custom‐written MATLAB (MathWorks, Natick, MA) script (published in Young et al.^41^) was adapted and modified to facilitated velocity calculation by manually entering frame numbers of the animal crossing between reference points. The MATLAB script adjusted forces for direction of travel, orientation, and limb (i.e., forelimbs vs. hindlimb). Only trials with distinct forelimb and hindlimb footfalls were analyzed. Standardized force signs reflected applied force by the animal, with vertical forces toward the force plate being positive, fore‐aft forces split into negative braking and positive propulsive forces, and mediolateral forces exhibiting negative medial and positive lateral orientations. Forces were filtered using a low‐pass Fourier filter at 15 Hz. Peak fore‐aft, vertical, and mediolateral forces were extracted from each hindlimb single limb footfall. All peak force data were normalized to the animal's body weight (%BW) for interindividual statistical comparison.

The net impulse were calculated in the fore‐aft plane to differentiate the overall role of the fore‐ and hindlimbs during walking by taking a sum of the braking and propulsive impulses.^42^ Positive net impulses indicate propulsive limbs, whereas negative values signify net braking limbs, and values approximating zero denote single limb contact forces with roughly equal braking and propulsive impulses.

All data were analyzed using R (R Core Team, 2021) with packages “ARTopl,” “lme4,” and multcomp. Data were first evaluated for normality and heteroscedasticity with Shapiro–Wilk and Levene's tests evaluated data set normality,^43^ before rank‐transforming to satisfy assumptions for statistical analyses. A series of linear mixed effect models assessing the impact of mice type (WT vs. KO), age (2 vs. 4 months), foot (left vs. right), and limb (forelimb vs. hindlimb) on the peak forces in each plane (fore‐aft, normal, and mediolateral) were created. Speed, well‐known to influence force data magnitude,^44^ was incorporated in each model as fixed effect. Individual idiosyncrasies were controlled for by including individual as a random effect in each model. All data necessary for statistical replication can be found in Supplemental Table S1.

### 2.5 Ex vivo isometric contractile analysis

Isometric force production and fatigue resistance of the extensor digitorum longus (EDL) (*n* = 10/group) and soleus (*n* = 8/group) muscles were evaluated ex vivo, following previously established protocols.^45^, ^46^, ^47^, ^48^ Muscles were surgically excised and mounted to a force transducer apparatus (Aurora Scientific Inc., 407B) using 5–0 polyglactin suture ligatures attached to each tendon. The muscles were submerged in a bath of oxygenated Krebs Ringer Buffer (KRB—[mM] 118 NaCl, 4.7 KCl, 1.3 NaH_2_PO_4_, 2.5 CaCl_2_, 25 NaHCO_3_, 1.2 MgSO_4_, 10 Glucose, 0.03 α‐tubocurarine chloride; pH 7.4) maintained at room temperature (25°C). After a 5‐min equilibration period, the optimal resting length (L_0_) was determined by stimulating the muscles with a single 1 Hz twitch every 30 s, adjusting the muscle length to maximize force output. Supramaximal stimulations were induced by a bi‐phasic stimulator (Aurora Scientific, 701C) and delivered through parallel platinum electrodes (~9 mm apart) surrounding the muscle in the bath solution.

Upon determining L_0_, muscles were stimulated every 60 s at varying frequencies—10, 20, 60, 100, 140, 180, and 220 Hz for EDL, and 10, 20, 60, 100, 150, and 200 Hz for soleus—using 500 ms duration pulses to generate force‐frequency curves. Muscles rested for an additional 60 s before undergoing a 5‐min fatigue resistance protocol (100 Hz every 1 s with 500 ms duration, totaling 300 contractions). After measuring L_0_ with digital calipers, proximal and distal tendons were trimmed using micro scissors, and the muscles were blotted to remove excess KRB before determining muscle wet weight. Force output data were collected and analyzed using Dynamic Muscle Control LabBook 610A and Dynamic Muscle Analysis 611A software, respectively (Aurora Scientific Inc.). Absolute normal force (mN) was normalized to muscle mass and physiological cross‐sectional area (PCSA) to calculate muscle specific force (mN/mm^2^). PCSA was estimated using previously described equations, accounting for the density of mammalian skeletal muscle^49^ and myofiber length/whole muscle length ratios of 0.45 and 0.7 for the EDL and soleus, respectively.^50^

### 2.6 Histology

Soleus muscles (*n* = 3/group) were analyzed using immunofluorescence, following standard protocols established and routinely employed in our laboratories.^21^, ^22^ Based on the findings from *ex vivo* contractile tests, we focused our analysis on the soleus muscle. Muscles were embedded in optimal cutting temperature (OCT) medium and rapidly frozen in liquid nitrogen. Cryosections (8 μm) obtained from the muscle midbelly were subjected to immunostaining with the following primary antibodies: myosin heavy chain (MHC) type I (DSHB‐#A4.840, 1:25), MHC Type IIa (DSHB‐#SC‐71, 1:200), and MHC type IIb (DSHB‐#10F5, 1:25). Alexa Fluor 488 secondary antibodies (#A2‐1121, Life Technologies) were employed for MHC type I at 1:200, MHC type IIa at 1:500, and MHC type IIb at 1:200. Tertiary antibodies (ab6907, Abcam) were used at a 1:400 dilution for MHC type I and MHC type IIb. Type IIx fibers remained unstained. Cross‐sections were imaged using an Axio Scan.Z1 (Carl Zeiss Microscopy, München, Germany) microscope and analyzed with Zen Blue 3.6 software (Carl Zeiss Microscopy GmbH).

### 2.7 Data analyses and statistics

All data are presented as the mean ± SEM unless stated otherwise. Significant difference between genotype groups determined by unpaired Student *t*‐test, ANOVA, or linear mixed model. GraphPad Prism (Version 9.4.0) and R (R Core Team, 2021) was used for statistical analysis and for graphical representations.

## 3 RESULTS

### 3.1 Gene expression

**FIGURE 1:**
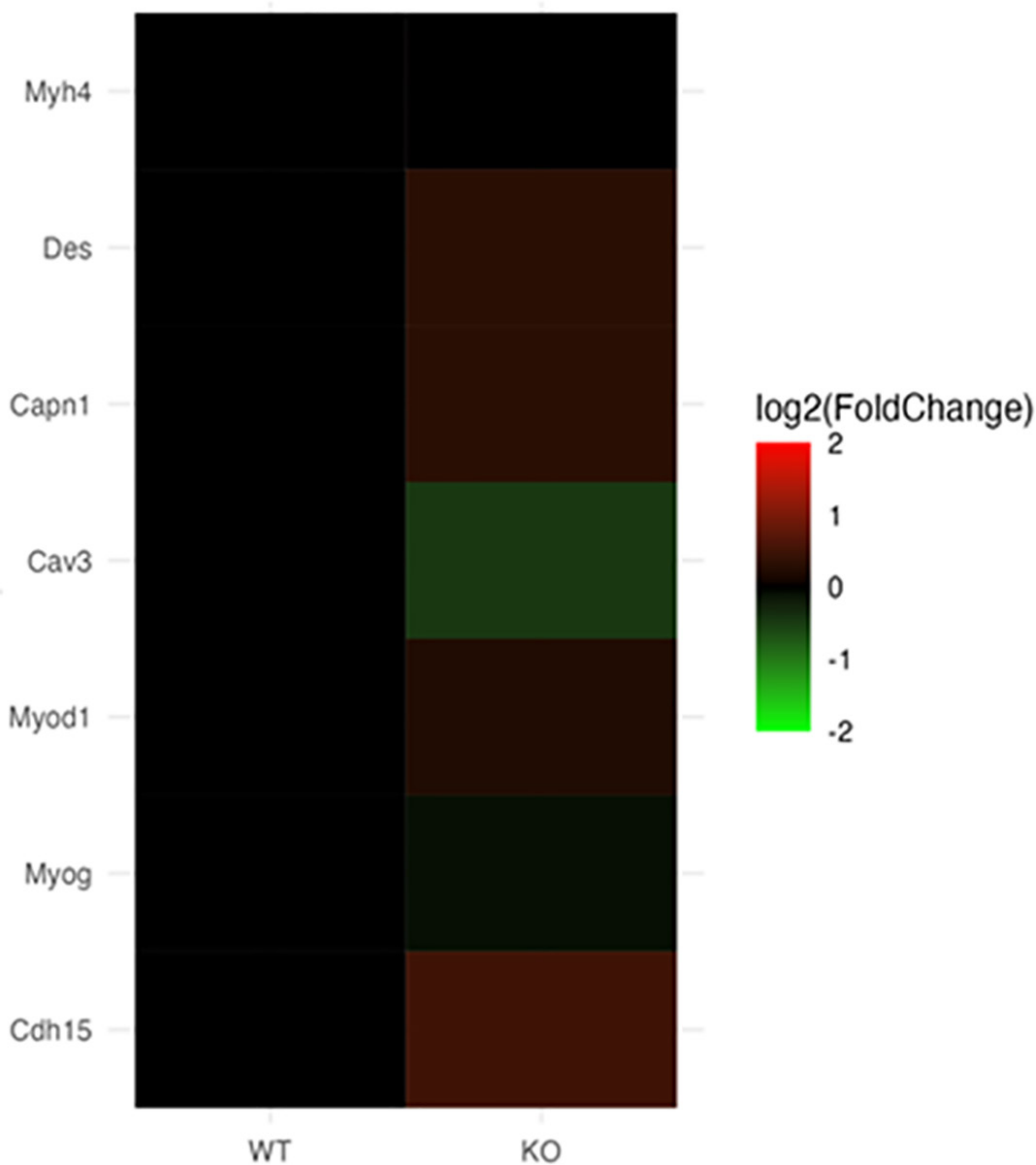
Differential mRNA expression between 2‐month‐old WT and KO male mice. Heatmap of selected myogenic differentiation and fusion genes.

**FIGURE 2:**
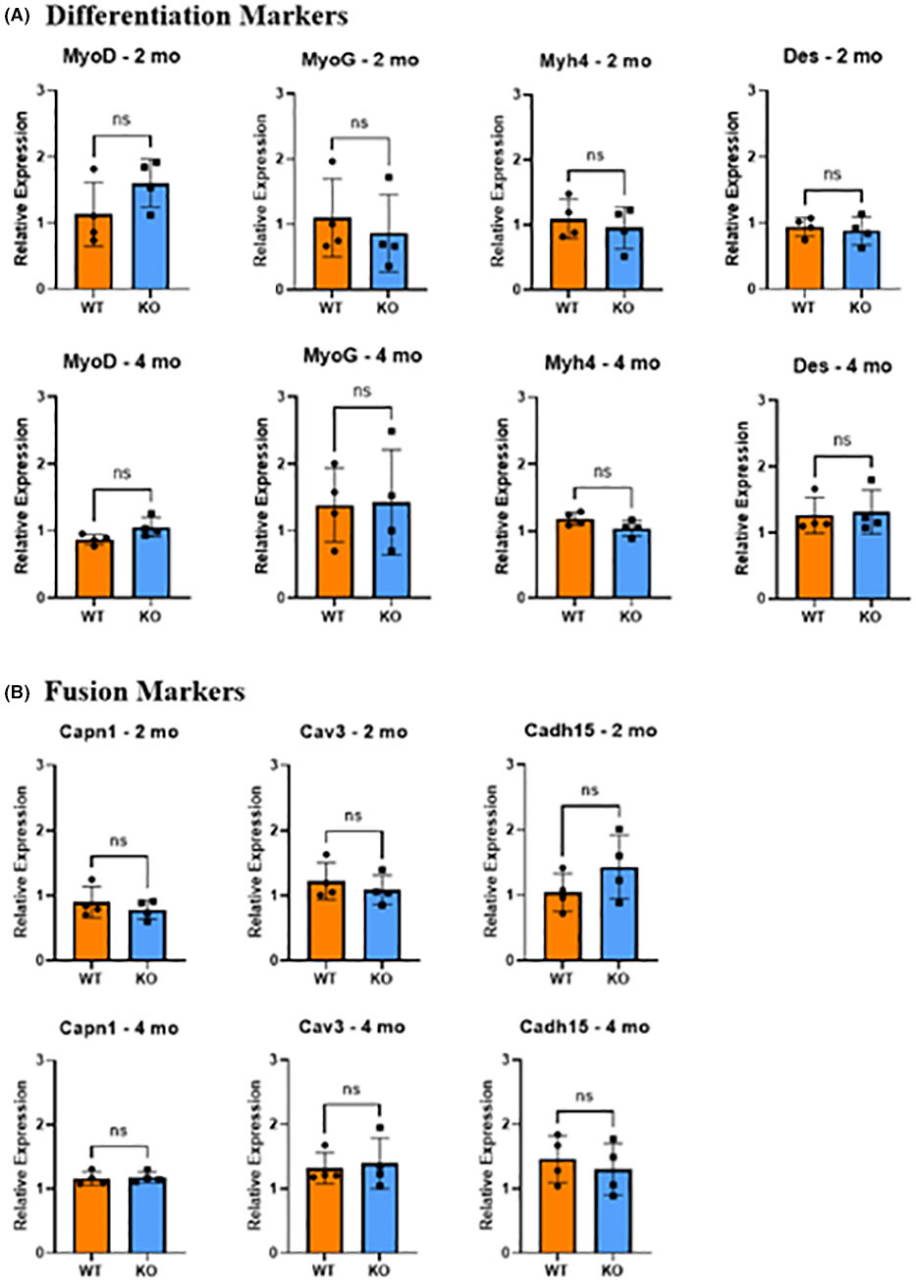
Evaluation of differentiation and fusion gene expression. (A) Expression levels of differentiation factors, including myoblast determination protein 1 (MyoD), myogenin (MyoG), myosin heavy chain 4 (Myh4), and desmin (Des) at 2 and 4 months. (B) Expression of fusion factors, calpain 1 (Capn1), caveolin 3 (Cav3), and cadherin 15 (Cadh15) at the same time points (*n* = 4/group, ns = non‐significant; unpaired *t*‐test). Error bars indicate SD derived from four independent PCR runs.

Microarray analyses revealed that the expression of specific marker genes (MyoD, MyoG, Myh4, Des, Capn1, Cav3, and Cadh15) involved in myogenic differentiation and fusion was slightly altered (either up or downregulated) between the WT and KO mice as indicated by the heat map (Figure 1). Subsequently, Q‐PCR expression data showed that there were no significant differences in the expression of any of these myogenic differentiation and fusion markers between the WT and KO mice (Figure 2).

### 3.2 Forelimb grip strength

**FIGURE 3:**
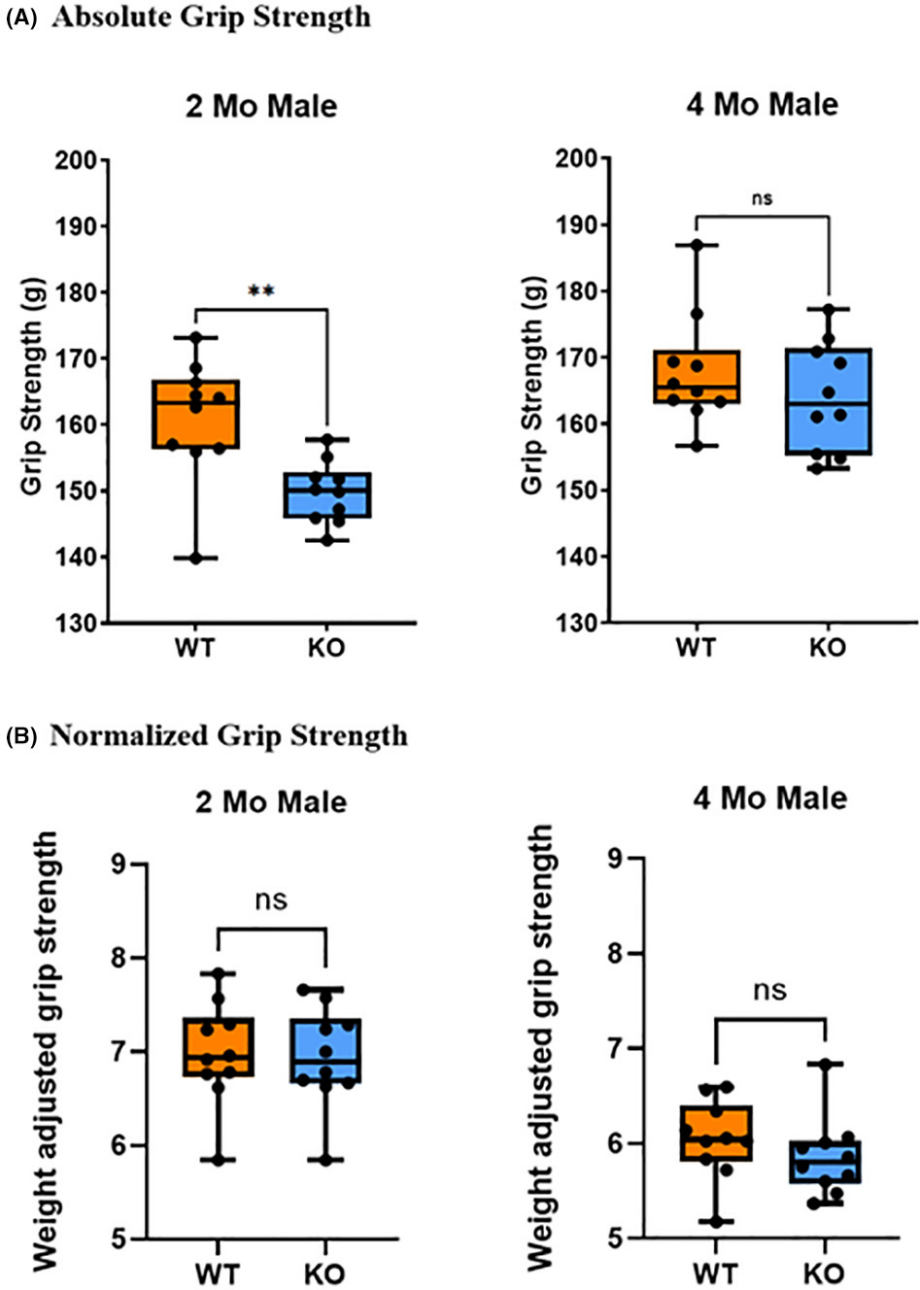
Determination of forelimb grip strength. Each trial was conducted six times at 1‐min intervals. The experimenter was blinded to the age and genotype of the mice being tested. (A) Raw grip strength data of 2‐ and 4‐month‐old male KO mice and WT counterparts. (B) Normalized grip strength by body weight (*n* = 10/group, **p < 0.01; Welch's *t*‐test).

Initially, the grip strength was measured in terms of its absolute value and at 2 months of age, male KO mice exhibited significantly (*p* < 0.01) lower grip strength in average of 15 g (~10%) than their WT counterparts (Figure 3A). This difference persisted in 4‐month‐old male mice but was not statistically significant (Figure 3A). However, when we normalized grip strength values to account for weight, no significant differences were observed in both age groups (Figure 3B).

### 3.3 Single limb force

**FIGURE 4:**
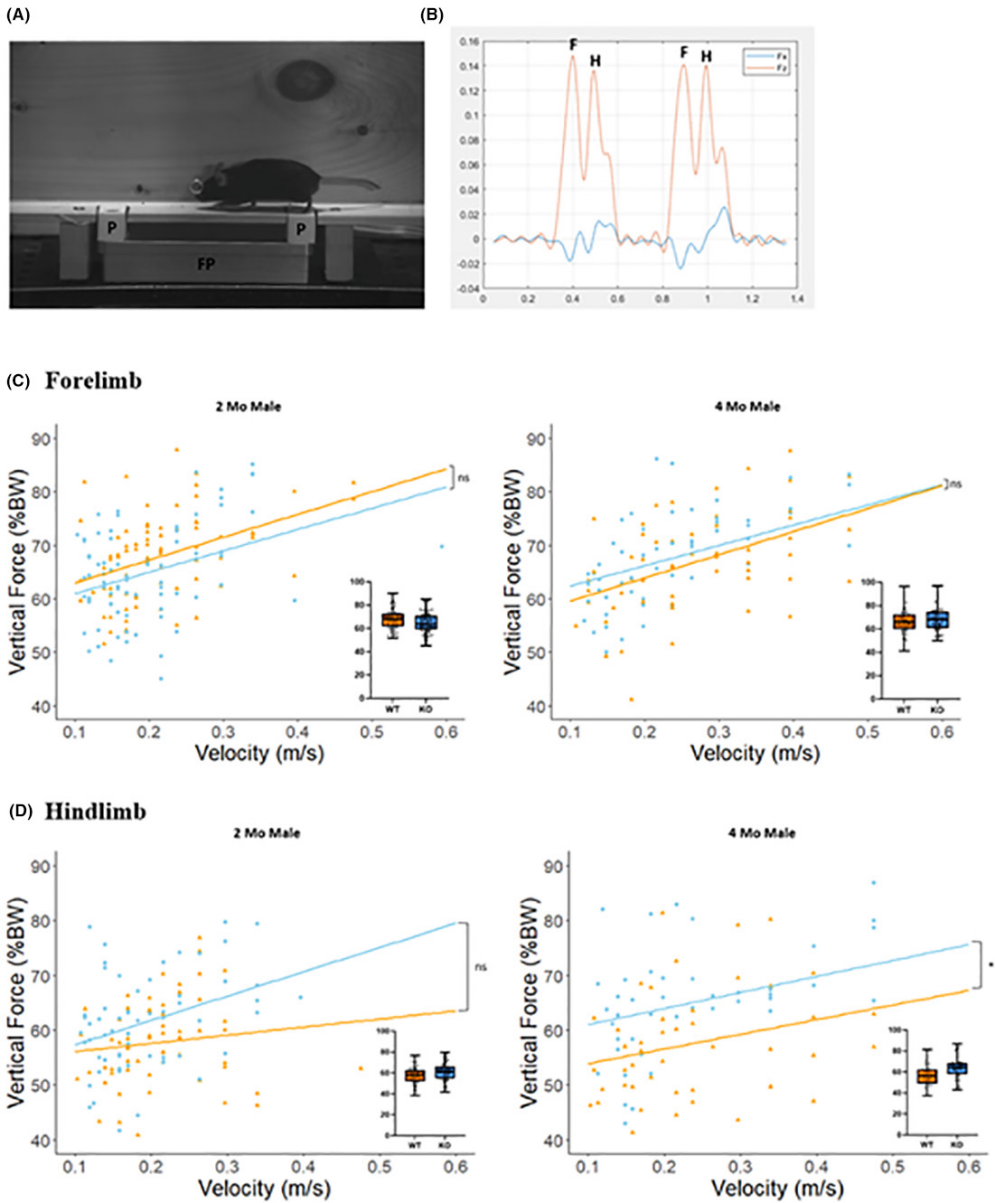
Single limb force analysis. (A) Experimental setup wherein a mouse traverses a runway fitted with two platforms “P” that are integrated with a force plate “FP”. (B) Corresponding force plate outputs, characterizing two forelimb “F” and two hindlimb “H” forces. “Fx” is fore‐aft plane and “Fz” is vertical plane. (C) and (D) delineate the vertical forces exerted by the forelimb and hindlimb, respectively, at 2 and 4 months of age. Boxplots illustrate the distribution of vertical forces. Each data point in linear graphs signifies an individual trial (*n* = 5/group, *p < 0.05; linear mixed model).

In the 2‐month‐old cohort, we analyzed a total of 164 forelimb trials (KO = 82; WT = 82) and 131 hindlimb trials (KO = 67; WT = 64). In the 4‐month‐old cohort, our sample included 126 forelimb trials (KO = 61; WT = 65) and 97 hindlimb trials (KO = 51; WT = 46). An image of a mouse on the force plate is shown in the Figure 4A. The corresponding force plate outputs, characterizing two forelimb “F” and two hindlimb “H” forces with Fx representing fore aft plane and Fz is vertical plane is shown in Figure 4B.

Among the five directional forces measured, only the vertical force demonstrated significant differences in mixed linear model, with the other forces showing no statistical significance (Table 2). A temporal shift was noted in the forelimb vertical force at the 2 months, where WT mice (67.88 ± 8.33 %BW) exhibited a higher forelimb force by approximately 3% than KO mice (64.62 ± 8.88 %BW). However, at the 4 dmonths, the average vertical force generated by the forelimbs of WT mice (66.70 ± 10.53 %BW) showed a marginal decrease of about 1%–3% compared to KO mice (68.11 ± 10.16 %BW) at a consistent velocity (Figure 4C), a difference that did not reach statistical significance. On the other hand, the hindlimb vertical force of KO mice (60.76 ± 8.91 %BW) consistently exceeded that of WT mice (56.95 ± 8.20 %BW) by ~2–15% at higher velocities at 2 months, although the difference was not statistically significant. At 4 months, the KO mice's hindlimb vertical force (64.03 ± 9.96 %BW) was approximately 10% greater than that of the WT mice (56.64 ± 10.61 %BW) with statistically significance (*p* < 0.05) (Figure 4D).

**TABLE 2:** Statistical maps the significance (*p*‐value) of five directional forces relative to gravity for (A) forelimb. (B) Hindlimb.

(A)					
Forelimb	Fore	Aft	Medial	Lateral	Vertical
2 mo	0.0754	0.7336	0.8883	0.2635	0.1904
4 mo	0.5881	0.2543	0.8702	0.2180	0.0824

(B)					
Hindlimb	Fore	Aft	Medial	Lateral	Vertical
2 mo	0.5817	0.7252	0.7940	0.0568	0.0797
4 mo	0.4671	0.7794	0.9420	0.9477	0.0135*

*Note*: *n* = 5/group.

* *p* < 0.05, linear mixed model.

### 3.4 Ex vivo isometric contractions

**FIGURE 5:**
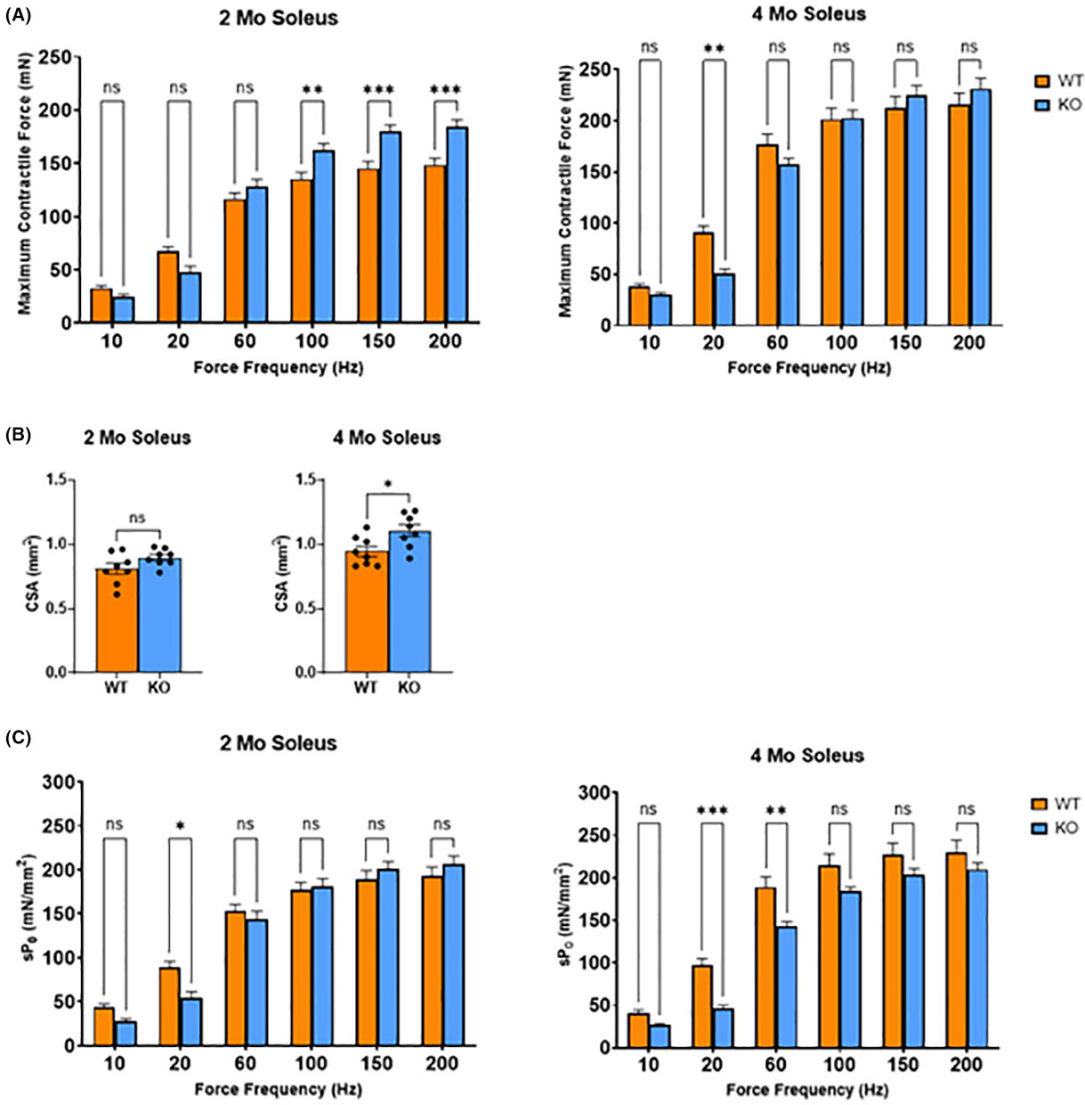
Evaluation of ex vivo isometric contractile force. (A) Absolute isometric force production of the soleus muscle across various frequencies for the 2‐ and 4‐month WT and KO mice. (B) Physiological cross‐sectional area (PCSA) comparison between WT and KO (*n* = 8/group, *p < 0.05; Mann–Whitney test). (C) Specific isometric force production of the soleus muscle, normalized by PCSA, at the corresponding frequencies. Upon normalization of the contractile force using PCSA, the 2‐month‐old KO mice showed a significant difference in specific force at 20 Hz, whereas the 4‐month‐old KO mice revealed a more marked decrease at 20 and 60 Hz (*n* = 8/group, ***p < 0.001; **p < 0.01; *p < 0.05; two‐way ANOVA; Mann–Whitney test). Data represented by mean ± SEM.

The results of the ex vivo isometric contractile tests revealed significant discrepancies exclusively in the soleus muscle (Figure 5), while the EDL muscle did not show any significant difference across force frequency tests and fatigue tests in both age groups (Supplementary Data Figures 1 and 2). The soleus muscle of the 2‐month‐old KO mice exhibited a significantly higher absolute contractile force at high frequencies by approximately 30–40 mN (~20%–24%) (*p* < 0.01 at 100 Hz; *p* < 0.001 at 150 and 200 Hz) (Figure 5A). Conversely, at 20 Hz, 4‐month‐old KO mice presented a significantly lower contractile force by 20 mN (~55%) as compared to their WT counterparts (*p* < 0.01) (Figure 5A).

PSCA was marginally higher in KO mice by ~0.1 mm^2^ (~10%) on average at 2 months, albeit nonsignificant. This difference extended to at 4 months, with the KO mice showing a significantly larger by ~0.3 mm^2^ (~17%) (*p* < 0.05) (Figure 5B). Upon normalizing the contractile force using the PCSA, the specific forces of the 2‐month‐old mice showed a significant difference by approximately 30 _S_P_0_ (mN/mm^2^) only at 20 Hz, where KO mice generated about 40% lower force than WT mice (*p* < 0.05). The 4‐month‐old mice, however, demonstrated a more pronounced difference by approximately 20–50 _S_P_0_ (mN/mm^2^) at 20 Hz (*p* < 0.001) and 60 Hz (*p* < 0.01), with KO mice again exerting about 50% and 20% lower force than WT mice, respectively (Figure 5C).

**FIGURE 6:**
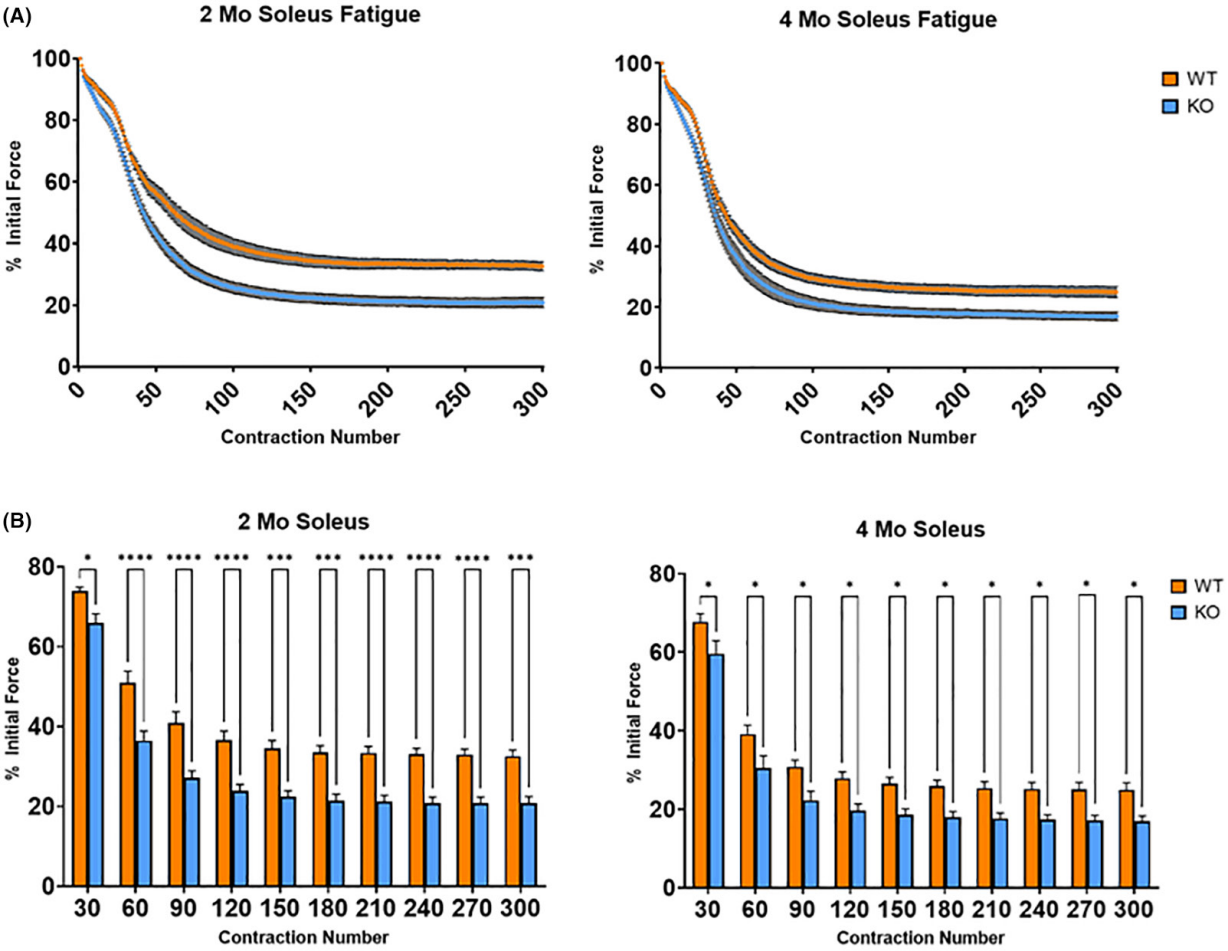
Isometric fatigue test. (A) Fatigue curve represents the performance of the soleus muscle during 300 contractions executed over a period of 5 min for the 2‐ and 4‐ month WT and KO mice. (B) The percent initial force produced is displayed for specified contraction numbers. The protocol revealed significant variations between the KO and WT mice in both age groups (*n* = 8/group, ****p < 0.0001; ***p < 0.001; *p < 0.05; two‐way ANOVA). Data represented by mean ± SEM.

The fatigue resistance protocol involving 300 contractions over a 5‐min span revealed significant differences between KO and WT mice across both age groups. At 2 months, the KO mice exhibited a significantly diminished percentage of ~20% lower initial force at the 30th contraction (*p* < 0.05) (Figure 6A). This significant difference increased markedly from the 60th contraction onwards up to the 300th contraction. Similarly, the 4‐month‐old KO mice demonstrated a ~10% reduction in the percentage of initial force from the 30th contraction onwards to the 300th contraction (*p* < 0.05) (Figure 6B).

### 3.5 Histology

**FIGURE 7:**
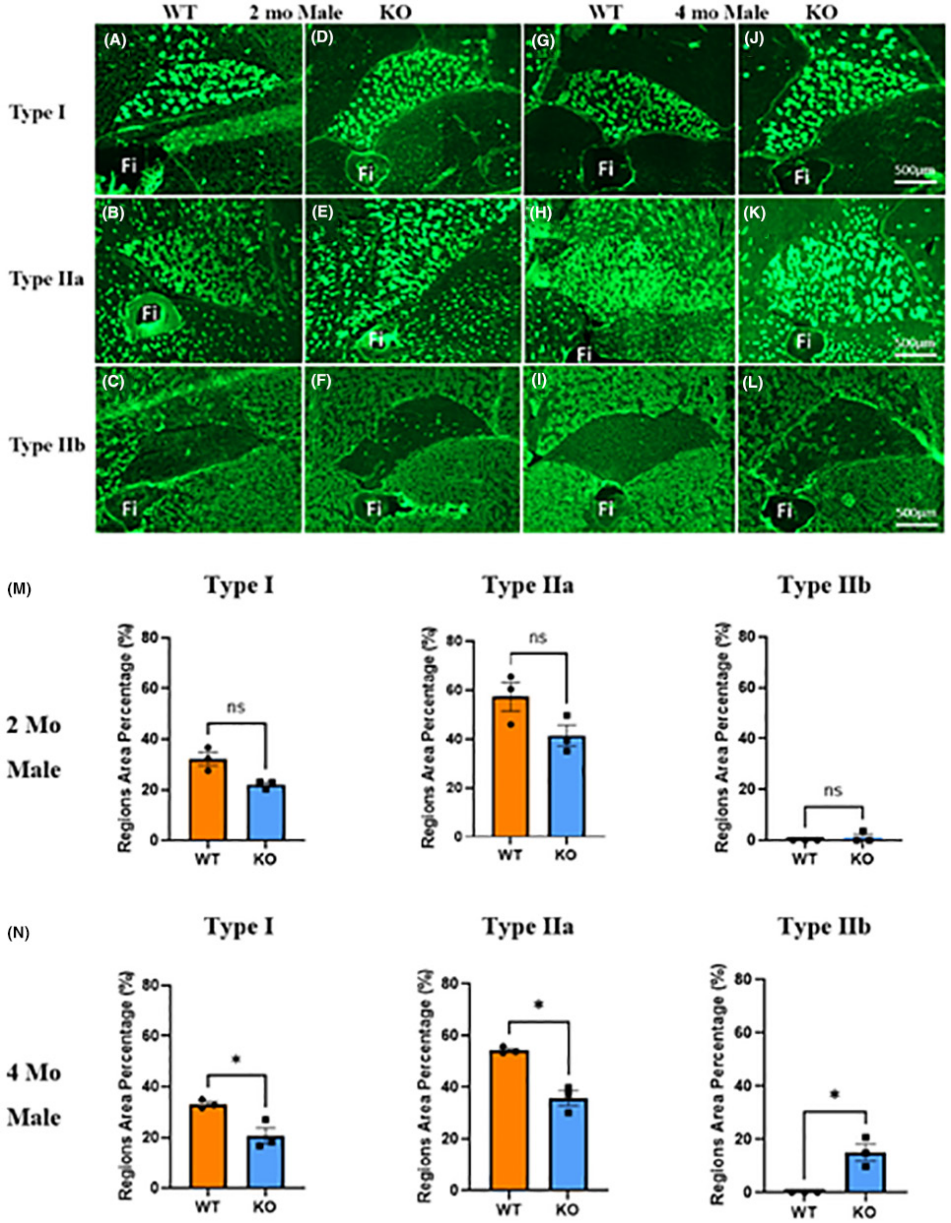
Skeletal muscle fiber typing. Immunofluorescence images of the soleus muscle taken at 2 and 4 months of age (A–L), indicating the various fiber (Type I, Type IIa, and Type IIb) type compositions. (Fi = Fibular). (M) Quantitated analysis of stained sections for each muscle fiber type at 2‐months and (N) 4 months (*n* = 3/group, *p < 0.05; Welch's *t*‐test). Data represented by mean ± SEM.

Immunofluorescence analysis of the soleus muscles was employed to further investigate the structural implications of *Mustn1* deletion on skeletal muscle fiber type (Figure 7). At 2‐months, the KO mice demonstrated a trend for an ~8% and 18% reduction in the proportion of Type I and Type IIa fibers, respectively, as compared to the WT mice, although this difference was not statistically significant (Figure 7M). Notably, at 2 months, KO mice displayed the presence of Type IIb fibers, a subtype that was absent in the WT soleus muscle (Figure 7C,F,M). By the time the mice reached 4 months of age, the differences in fiber composition became statistically significant (Figure 7N). The KO mice exhibited a ~15% and 20% decrease in the proportion of Type I and Type IIb fibers, respectively (*p* < 0.05). Further, a significant increase of Type IIb fibers by ~15% was observed in the KO mice (*p* < 0.05), a subtype that remained absent in the WT soleus muscle (Figure 7I,L,N).

## 4 DISCUSSION

Building upon previous in vivo and in vitro data on *Mustn1* and its role in skeletal muscle development, myogenic differentiation, and fusion,^11^, ^17^, ^18^, ^21^, ^28^ our experiments aimed to further investigate these processes. Previous studies have shown that silencing of *Mustn1* culminates in abrogated myoblast differentiation and complete failure of myofusion in vitro,^21^ and the in vivo inhibition of *Mustn1* translation via RNA interference (RNAi) resulted in distinct malformations in *Xenopus*.^27^ Hu et al.^28^ also showed that *Mustn1* is an important regulator of proliferation and differentiation of chicken skeletal muscle satellite cells.

To elucidate the implications of *Mustn1* ablation in skeletal muscle development, we conducted gene expression analyses (both microarray and Q‐PCR) on RNA samples extracted from the gastrocnemius muscle of 2‐month‐old and 4‐month‐old WT and KO mice. The data obtained from these experiments confirmed that *Mustn1* expression was negligible in both 2‐month‐old and 4‐month‐old mice, thereby validating the successful ablation of *Mustn1* in the skeletal muscle, as previously reported.^29^ However, the KO mice did not present any distinct phenotypic skeletal muscle differences as we previously reported other than temporal weight differences.^29^

While silencing of *Mustn1* using RNAi in vitro led to significant decreases in the expression of all myogenic differentiation and fusion markers, in this in vivo study, no major or significant changes were observed. This discrepancy could be due to several factors. One obvious difference is in the fact that the Liu et al. study was conducted in vitro with a cell line (C2C12), while this study was based in vivo with mice. Also, although the Q‐PCR data from our previous study confirmed clear downregulation of *Mustn1* in skeletal muscle, a minimal amount of *Mustn1* expression persisted.^29^ Considering that *Mustn1* is not solely expressed by skeletal muscle but is also produced by cartilage, bone, and tendon,^9^ it is plausible that other cell types may compensate for *Mustn1* production when downregulation occurs in one cell type.

The conversion of fibroblasts to myoblasts in the presence of myoD has been documented in several studies.^51^, ^52^, ^53^ In the 2‐month‐old KO male mice from our study, myoD levels were slightly elevated, although this increase was not statistically significant. Further, the specific stage at which this may occur and thus affecting *Mustn1* expression remains unknown. Based on our data, which show a mitigation of observed differences in adulthood, we hypothesize that any such changes probably occur at earlier developmental times and across different cell types but this remains to be determined.

Forelimb grip strength was conducted to assess the impact of *Mustn1* ablation on muscle strength. Based on the findings of Takeshita et al.,^31^ grip strength in adult mice remains stable from 3 to 18 months, indicating that 4‐month‐old mice serve as a suitable model for representing adulthood. In initial assessments of forelimb grip strength, KO mice exhibited a significant reduction in absolute strength at 2 months, but dissipated at 4 months. Additionally, when adjusted for body weight, the difference in grip strength between WT and KO mice were no longer significant indicating that the observed difference at 2 months is most likely attributable to the significant disparity in body weight between the WT and KO mice from 1 to 3 months of age. We previously showed that the KO mice presented with substantially lower body weight as compared to their WT counterparts until the age of 3 months. Beyond the 4‐month mark, this difference in body weight became nonsignificant.^29^ Further, in the modified grip strength tests, the grip bar was positioned vertically, contrast with the traditional horizontal setup.^31^ Thus, the weight difference between the WT and KO mice could possibly serve as a confounding factor in this configuration due to the gravitational pull acting on the mice as they grasped the grip bar, especially as body weight is known to be a confounding variable in grip strength tests.^54^ Lastly, previous muscle and bone studies also normalized by weight and in some cases significant differences no longer remained.^55^, ^56^

We also postulated that changes in skeletal muscle, stemming from *Mustn1* ablation, might potentially lead to observable alterations of limb loading dynamics. Among the three planes of force, only the vertical force exhibited a significant difference at 4 months of age, demonstrating a specific influence of *Mustn1* ablation on this force vector. Previous studies have demonstrated that hypermuscular mice with myostatin‐deficiency indicated changes in locomotion by affecting transverse ground force.^57^ Considering our findings, we hypothesize that the increased PCSA of the soleus muscle could potentially be responsible for the observed change in vertical forces. Interestingly, the data also revealed a temporal shift in the average vertical forces exerted by the forelimb and hindlimb in KO mice compared to WT mice, both at the 2‐ and 4‐month time points. This temporal shift in force profiles was specific to each limb, emphasizing the intricate and evolving impact of *Mustn1* ablation on the locomotive biomechanics in mice.

As suggested by Schmitt et al.,^57^ ground reaction forces generated during locomotion can influence the loading environment for long bones and possibly muscles, leading to changes in bone size and strength. Considering that *Mustn1* is exclusively expressed in the musculoskeletal system and was initially discovered during a bone repair study,^9^ it would be valuable to investigate how the increased PCSA of the soleus muscle affects bone size and strength. This observation highlights the complex role of *Mustn1* in muscle function and suggests the possibility of compensatory or adaptive mechanisms at play in response to its ablation.

To delineate the potential effects of *Mustn1* ablation on skeletal muscle contractile force, we strategically selected the soleus and EDL muscles for analysis due to their representative nature for Type I and Type II muscle fibers, respectively.^58^ Our findings from the isolated ex vivo skeletal muscle isometric contraction tests revealed that *Mustn1* ablation did not elicit any discernible effect on the EDL muscle. However, the soleus muscle did was affected in the KO mice. Given that the soleus, a major plantar flexor, is essential for activities such as walking, running, and climbing, an increase in Type IIb fibers in this muscle could be contributing to the observed elevation in single limb vertical force during walking and the higher absolute contractile force in the KO mice during the ex vivo isometric contraction test. Furthermore, the decrease in Type I slow‐twitch, fatigue‐resistant fibers could elucidate the observed higher fatigue susceptibility and lower contractile force during the fatigue test in KO mice.

Minchew et al. (2022)^59^ reported that various mouse strains display distinct muscle fiber composition, that affects isometric contractile force and fatigue test outcomes. This suggests the differences we observed may be attributed to varied fiber compositions in the soleus muscle. Interestingly, while Minchew et al. (2022) noted differences in both EDL and soleus muscles across mouse strains, we only discerned variations in the soleus. We hypothesize that this discrepancy may be related to the role of *Mustn1* in primarily regulating Type I myofiber development during myogenesis, with Type II fibers appearing later.^60^ Past research has shown that alterations in specific genes can precipitate changes in muscle fiber types, often resulting in increases in Type IIb fibers.^61^, ^62^, ^63^, ^64^, ^65^ These investigations underscore the role certain genes play in modulating fiber composition within skeletal muscle, with the consequence of traditionally oxidative muscle types switching to adopt more glycolytic characteristics. Furthermore, certain genes have been found to induce skeletal muscle hypertrophy and hyperplasia at the same time.^65^

While the exact mechanisms of *Mustn1* involvement in myogenesis remain to be fully elucidated, current data suggest a more substantial impact on Type I muscles. In support of this, McKenzie et al.^24^ demonstrated that aerobic exercise modulates *Mustn1* expression specifically in the soleus muscle, but not in the gastrocnemius, which comprises of a mix of Type I and II muscle fibers. Our findings further imply a specific relationship between *Mustn1* and Type I fibers, potentially explaining the reduced composition of Type I fibers observed at both 2 and 4 months in the KO mice. Further, considering that Type II fibers develop from Type I,^60^ the decrease in the regional area percentage of Type IIb fibers could be a downstream effect of diminished Type I fibers. Yet, the role of *Mustn1* in the maturation or transition of these muscle fibers from Type I remains speculative. Interestingly, our results showed KO mice generating Type IIb fibers when their WT counterparts did not, hinting at a possible compensatory mechanism to offset lower Type I fiber counts. The question remains, however, why a Type I fiber would evolve into a Type IIb instead of Type IIa, providing an interesting direction for future research.

This lower composition of Type I fibers in KO mice could also potentially explain their lower body weight observed during the 1–3 month age span.^29^ Although their body weight was significantly lower at 2 months, the KO mice exerted a higher, though non‐significant, vertical force in the forelimb. This translates into a higher percent body weight (%BW) force, despite the absolute grip strength being significantly lower in the KO mice. By 4 months, when the body weight differences became nonsignificant, the forelimb vertical force exerted by the KO mice decreased compared to the WT mice.

In line with previous findings,^66^ only a small fraction of forelimb muscles in mice expressed Type I myosin heavy chain fibers, constituting less than 6% of these muscles. This low prevalence might explain the lack of noticeable effects of *Mustn1* ablation in the forelimb, in contrast to its clear influence on the hindlimb. By 4 months, the observed increase in Type IIb fibers in the hindlimb could account for the elevated vertical force. According to Schiaffino & Reggiani,^58^ contractile force peaks in the order of Type I < IIa < IIx < IIb, with Type IIb fibers exhibiting the highest contractile force across all mammalian species and muscle types. Noteworthy, a clear trend was observed in our experiments, not just between WT and KO, but noting differences between the 2‐ and 4‐month age groups. This aligns with our previous data showing changes in weight and glucose tolerance between these two age groups.^29^ Historically, it has been established that slow oxidative Type I fibers have a higher glucose uptake capacity than fast glycolytic Type II fibers^67^, ^68^, ^69^ and that insulin sensitivity can also influence glucose uptake.^70^, ^71^, ^72^ The exact mechanism and pathway responsible for the enhanced glucose tolerance in the 2‐month‐old KO mice remain to be fully elucidated and warrant further investigations.

For a more detailed and complete understanding of the role and function of *Mustn1* in skeletal muscle, future experiments should explore the effects of *Mustn1* ablation on muscle repair and regeneration, the influence of exercise, and the impact on other muscle groups with high Type I fiber composition. As a pan‐musculoskeletal cell marker, *Mustn1* has shown to have an effect on weight and glucose tolerance,^29^ as well as skeletal muscle function. In the light of these findings, further investigation into the molecular functions and regulatory pathways of *Mustn1* may hold potential for broad applications in the field of skeletal muscle physiopathology.

## AUTHOR CONTRIBUTIONS

Charles J. Kim conceived and designed the study, performed the research, acquired, analyzed, and interpreted the data, and played a major role in the writing of the manuscript. Chanpreet Singh, Marina Kaczmarek, and Madison O'Donnell significantly contributed to the research execution, as well as the acquisition and analysis of the data. Christine Lee and Kevin DiMagno also played key roles in the execution of the research. William Letsou analyzed the microarray data while Melody W. Young, Raddy L. Ramos, and Michael C. Granatosky primarily contributed to the interpretation of the data. Michael Hadjiargyrou, the senior author, collaborated in the conception and design of the study, interpretation of the data, edited the manuscript and provided overall supervision for the project. All authors were involved in drafting the manuscript and revising it critically for important intellectual content.

## DISCLOSURES

The authors declare no conflict of interest.

## Supporting information


Supplementary Figure 1. Please delete page 3 from this document.

Supplementary Table 1.

## Data Availability

The data that support the findings of this study are available from the corresponding author upon reasonable request.
