# Supplementary material for: Mustn1 ablation in skeletal muscle results in functional alterations
Source: FASEB Bioadv. 2023 Nov 15;5(12):541–57. doi: 10.1096/fba.2023-00082 (PMC10714068; doi:10.1096/fba.2023-00082)

Supplementary Data

Supplementary Figure 1. Ex Vivo Isometric Contractile Analysis. (A) Absolute isometric force production of EDL muscle at specified frequencies. (B) Physiological cross sectional area (PCSA) comparison between WT and KO (n – 10/group, ns = non-significant; Mann-Whitney test). (C) Specific isometric force production of the EDL muscle, normalized by PCSA, at indicated frequencies. (n = 10/group; 2-Way ANOVA). Data represented by mean ± SEM.

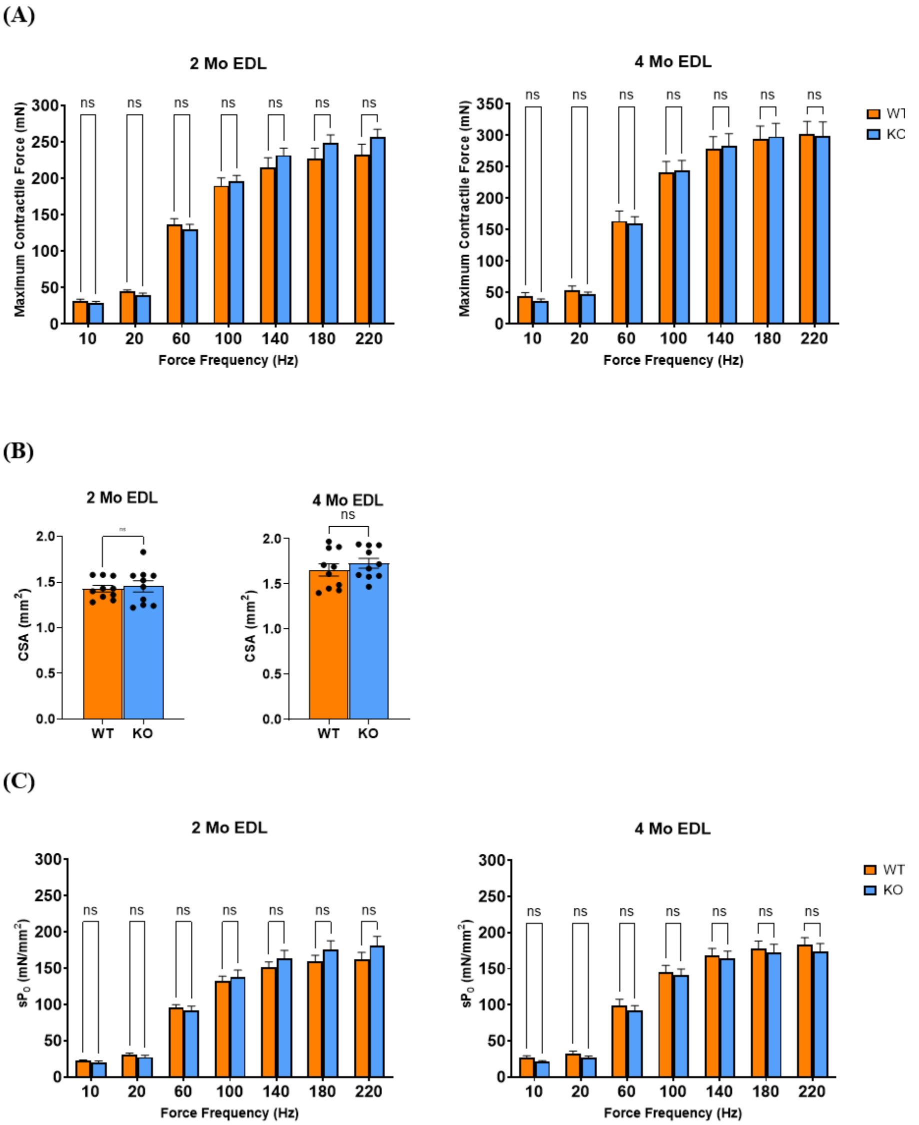

Supplementary Figure 2. Isometric Fatigue(A) Fatigue curve of EDL muscle depicting 300 contractions over a 5-minute period. (B) Percent initial force produced at specified contraction number. (n = 5/group; 2-Way ANOVA). Data represented by mean ± SEM.

(A)

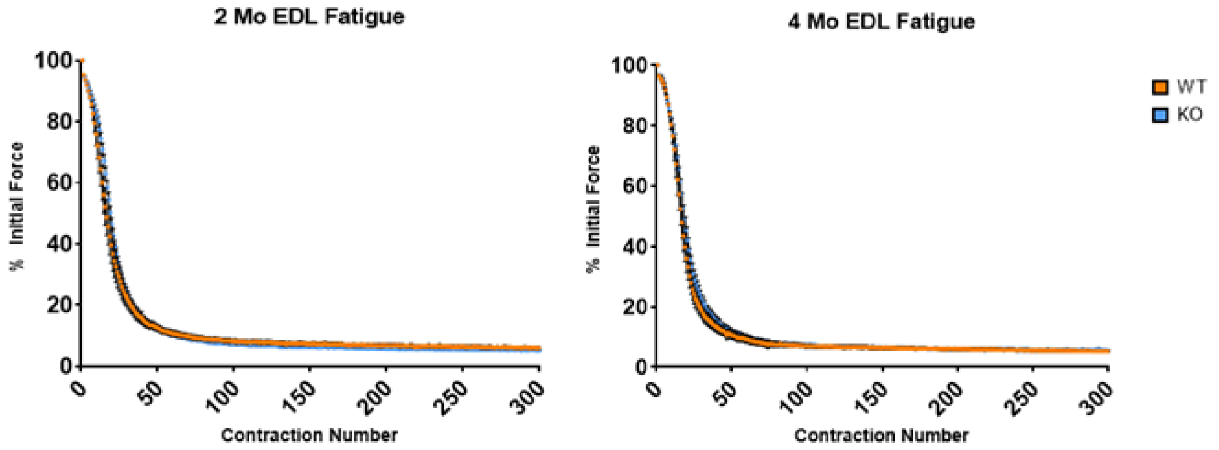

(B)

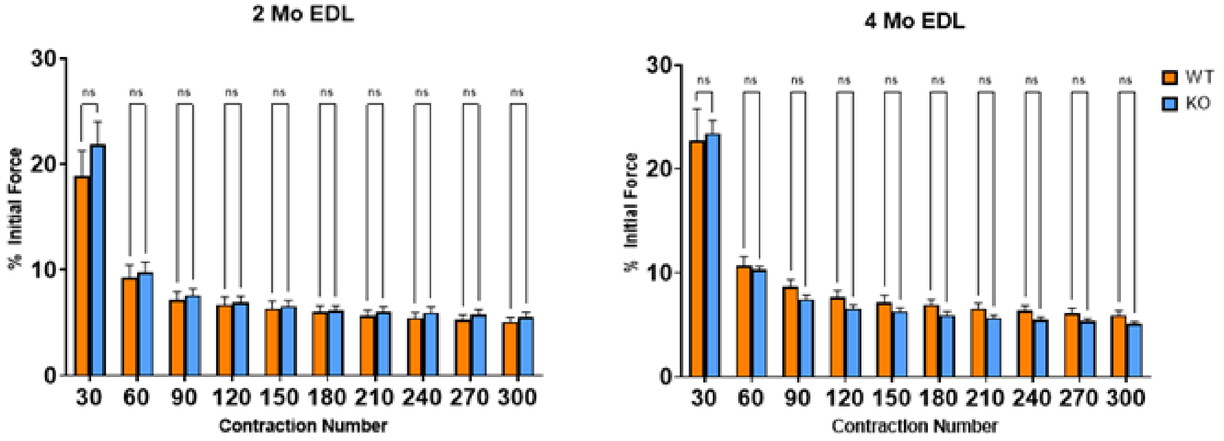

Supplement: Supplementary file 1 — Supplementary Figure 1. Please delete page 3 from this document. [file FBA2-5-541-s002.pdf]
