# Supplementary material for: Mustn1 ablation in skeletal muscle results in functional alterations
Source: FASEB Bioadv. 2023 Nov 15;5(12):541–57. doi: 10.1096/fba.2023-00082 (PMC10714068; doi:10.1096/fba.2023-00082)
Supplement: Supplementary file 2 — Supplementary Table 1. [file FBA2-5-541-s001.pdf]

Supplementary Table 1. Single Limb Force Plate Data. Standardized force signs reflected animal-applied forces, with vertical (“Z”) forces toward the force plate being positive, fore-aft forces split into negative braking (“X1”) and positive propulsive(“X2”) forces, and mediolateral forces exhibiting negative medial (“Med”) and positive lateral (“Lat”) orientations.

Month	Gt	Hit	elocity (m/	X1	X2	Med	Lat	Z
2	WT	forelimb	0.197917	-2.21551	2.576981	-1.94154	9.45817	70.09375
2	WT	forelimb	0.197917	-4.77508	4.709289	-2.63285	8.201072	66.87137
2	WT	forelimb	0.169643	-14.6898	0.675981	-0.20946	10.02188	64.36501
2	WT	forelimb	0.182692	-14.2509	2.655745	-2.22733	8.549761	69.16438
2	WT	forelimb	0.215909	-7.39145	5.095588	-1.54541	6.426231	62.90098
2	WT	forelimb	0.263889	-26.1945	0.924248	-3.82209	12.49088	83.34502
2	WT	forelimb	0.169643	-35.3582	1.135325	-3.53089	13.47442	82.8285
2	WT	forelimb	0.263889	-11.376	2.669413	-3.25215	9.770687	81.19244
2	WT	forelimb	0.296875	-9.42437	9.424368	-6.92346	5.15249	67.56394
2	WT	forelimb	0.148438	-13.9348	1.457983	-2.83966	3.118798	67.93063
2	WT	forelimb	0.169643	-6.96755	0.397094	-1.06072	7.587713	57.90798
2	WT	forelimb	0.113095	-8.04882	7.038845	-3.5899	6.619109	81.78904
2	WT	forelimb	0.182692	-12.5399	6.923664	-2.65023	4.572375	69.17516
2	WT	forelimb	0.296875	-6.23353	1.865527	-4.62184	4.796209	62.27677
2	WT	forelimb	0.158333	-6.77643	0.763576	-3.77717	5.314875	67.75344
2	WT	forelimb	0.125	-4.05258	1.149027	-4.24801	10.63747	61.21812
2	WT	forelimb	0.395833	-26.5929	1.366277	-1.53368	16.33784	64.28265
2	WT	forelimb	0.139706	-9.59672	7.177301	-7.32959	7.141921	65.63734
2	WT	forelimb	0.098958	-7.13639	1.636749	-1.13209	9.022419	69.75288
2	WT	forelimb	0.053977	-7.66477	2.987951	-3.21914	6.403959	70.13825
2	WT	forelimb	0.148438	-6.42529	0.983841	-3.5086	5.266235	67.25068
2	WT	forelimb	0.197917	-7.8381	8.967606	-0.81576	8.196562	70.9384
2	WT	forelimb	0.182692	-12.9	2.803686	-2.5336	5.359758	60.65874
2	WT	forelimb	0.139706	-7.28472	4.945173	-0.95027	5.475303	61.93488
2	WT	forelimb	0.139706	-6.59477	0.918183	-5.10242	6.155244	51.51778
2	WT	forelimb	0.2375	-7.68958	4.061818	-2.34702	4.093645	73.0363
2	WT	forelimb	0.263889	-9.14176	3.341211	-3.63455	6.806351	69.64778
2	WT	forelimb	0.169643	-6.7571	0.751165	-0.10099	7.451412	68.97387
2	WT	forelimb	0.263889	-3.25591	5.686656	-3.0133	6.968974	67.11245
2	WT	forelimb	0.2375	-12.8567	0.300439	-1.08472	7.729875	87.71573
2	WT	forelimb	0.182692	-12.7932	1.135272	-2.47651	6.614604	70.03252
2	WT	forelimb	0.197917	-8.28141	2.665715	-5.48616	6.77045	73.80562
2	WT	forelimb	0.169643	-6.43309	4.072931	-2.54782	8.903455	55.70817
2	WT	forelimb	0.339286	-7.63547	9.198384	-1.1267	8.778318	71.33286
2	WT	forelimb	0.215909	-11.6611	0.727275	-1.88803	5.34033	73.11303
2	WT	forelimb	0.197917	-7.44442	4.35434	-6.05334	11.41496	67.12147
2	WT	forelimb	0.182692	-8.76162	0.766588	-1.45493	9.509626	60.11753
2	WT	forelimb	0.215909	-5.58757	0.07379	-4.1828	4.1828	69.27331
2	WT	forelimb	0.148438	-3.31455	7.811241	-3.18598	8.755747	60.60168
2	WT	forelimb	0.296875	-6.92896	6.212672	-6.11734	8.489052	71.54065

2	WT	forelimb	0.169643	-14.1946	2.811094	-1.19085	9.821838	54.11506
2	WT	forelimb	0.084821	-11.1564	1.479427	-0.66638	6.453412	59.99197
2	WT	forelimb	0.263889	-14.2399	2.401607	-1.89528	5.337467	63.5919
2	WT	forelimb	0.2375	-14.005	1.800867	-3.67942	8.680174	61.9257
2	WT	forelimb	0.169643	-19.4562	5.980095	-10.0453	10.04526	69.73854
2	WT	forelimb	0.148438	-11.8358	0.120032	-0.95725	8.699549	56.51489
2	WT	forelimb	0.169643	-6.17067	1.37586	-2.82972	9.487086	60.87955
2	WT	forelimb	0.2375	-10.6951	1.125579	-1.44924	13.62027	55.05919
2	WT	forelimb	0.339286	-6.24502	0.777607	-3.07328	5.599754	72.28021
2	WT	forelimb	0.148438	-12.4906	0.110097	-2.78201	5.752547	53.66149
2	WT	forelimb	0.107955	-8.12741	0.304778	-0.33033	7.775606	63.38105
2	WT	forelimb	0.139706	-13.8584	0.589719	-2.24125	9.286955	56.44739
2	WT	forelimb	0.263889	-21.0232	0.729186	-1.30866	6.289422	74.10753
2	WT	forelimb	0.098958	-3.64744	1.84372	-1.01705	5.694798	53.4452
2	WT	forelimb	0.197917	-13.3207	7.051685	-4.85929	5.114157	77.37831
2	WT	forelimb	0.158333	-7.36504	4.912881	-6.12658	11.68782	71.33975
2	WT	forelimb	0.197917	-6.99832	1.862053	-1.01566	9.303342	72.45122
2	WT	forelimb	0.197917	-23.6498	5.249159	-0.91056	11.37494	67.64832
2	WT	forelimb	0.197917	-15.0527	1.823974	-0.32735	8.071076	73.39401
2	WT	forelimb	0.169643	-16.771	2.313842	-0.40456	9.097983	72.55797
2	WT	forelimb	0.215909	-14.2056	4.202753	-0.99112	7.303706	68.77853
2	WT	forelimb	0.158333	-8.33812	1.664543	-1.86645	10.19814	68.22468
2	WT	forelimb	0.148438	-12.7248	0.912131	-0.23153	5.55099	61.84469
2	WT	forelimb	0.197917	-3.41081	2.191949	-1.11014	12.74908	66.50838
2	WT	forelimb	0.215909	-3.5369	5.032106	-2.14126	15.14994	68.51934
2	WT	forelimb	0.263889	-10.5898	3.003066	-0.81402	7.114302	56.41237
2	WT	forelimb	0.182692	-11.0565	1.896071	-5.51542	8.257606	58.43428
2	WT	forelimb	0.107955	-7.72477	7.2761	-5.32215	5.322147	74.52933
2	WT	forelimb	0.107955	-4.28569	4.880204	-4.5792	7.218391	59.62573
2	WT	forelimb	0.169643	-10.4307	0.317425	-2.77563	5.905928	53.76403
2	WT	forelimb	0.339286	-4.43849	9.419376	-1.59313	11.03629	72.05912
2	WT	forelimb	0.2375	-20.2016	3.301312	-5.71363	7.443983	77.35149
2	WT	forelimb	0.263889	-17.9791	0.837848	-1.06783	8.504385	73.07746
2	WT	forelimb	0.215909	-16.4804	2.984284	-1.56657	15.93344	65.71564
2	WT	forelimb	0.215909	-17.8374	2.842801	-6.84296	6.296851	71.75404
2	WT	forelimb	0.263889	-29.9999	1.295343	-2.78667	8.59652	79.17782
2	WT	forelimb	0.215909	-9.82153	4.722475	-4.1231	5.369236	57.90865
2	WT	forelimb	0.475	-29.8128	1.957183	-2.51936	5.451083	78.59921
2	WT	forelimb	0.395833	-21.4765	2.700942	-2.72155	9.500358	79.9678
2	WT	forelimb	0.339286	-15.3004	5.405571	-0.38222	21.01781	90.17882
2	WT	forelimb	0.263889	-35.2542	1.162298	-1.50678	12.87404	74.34294
2	WT	forelimb	0.475	-28.1395	1.008097	-1.06135	7.307431	81.61346
2	KO	forelimb	0.103261	-4.02516	3.954646	-1.82411	9.334884	71.98634
2	KO	forelimb	0.139706	-2.21598	6.177849	-3.04899	5.651715	66.69694
2	KO	forelimb	0.148438	-4.45228	3.436201	-4.23272	4.22128	65.42939

2	KO	forelimb	0.197917	-11.9992	2.606533	-3.73368	6.793123	67.2222
2	KO	forelimb	0.169643	-10.5687	0.257724	-7.75173	1.627456	64.17422
2	KO	forelimb	0.113095	-1.79644	5.036212	-4.37799	5.522677	63.95896
2	KO	forelimb	0.158333	-11.7297	0.188689	-2.14048	7.009769	57.50483
2	KO	forelimb	0.296875	-10.1323	1.2587	-4.70492	10.06161	80.50118
2	KO	forelimb	0.263889	-11.0612	0.830211	-2.21831	4.946361	68.46415
2	KO	forelimb	0.263889	-6.71146	1.229604	-2.86364	4.037116	67.43538
2	KO	forelimb	0.169643	-9.04152	2.965456	-15.4523	0.733272	52.02322
2	KO	forelimb	0.296875	-16.4904	0.257169	-0.73304	11.40003	78.87994
2	KO	forelimb	0.215909	-14.3563	0.650715	-1.58483	14.02196	66.24203
2	KO	forelimb	0.339286	-18.1882	2.415683	-6.4755	7.983732	76.14992
2	KO	forelimb	0.395833	-1.88184	1.985371	-0.89017	6.182172	59.69323
2	KO	forelimb	0.339286	-16.4687	1.213825	-1.07977	8.403819	83.20453
2	KO	forelimb	0.2375	-0.82543	8.131718	-0.34518	14.73743	53.91674
2	KO	forelimb	0.296875	-9.47363	0.393302	-2.29851	3.085383	62.5715
2	KO	forelimb	0.2375	-8.56364	4.79594	-2.00202	6.833937	73.48786
2	KO	forelimb	0.197917	-11.6049	5.41237	-2.38336	10.66245	66.43641
2	KO	forelimb	0.148438	-12.5999	0.717334	-1.33826	14.10297	71.25171
2	KO	forelimb	0.11875	-8.66881	1.940688	-4.22026	5.172668	59.36857
2	KO	forelimb	0.169643	-4.03393	12.03204	-3.5843	2.2612	53.83279
2	KO	forelimb	0.215909	-7.54471	1.70696	-0.97078	5.619544	62.25731
2	KO	forelimb	0.263889	-6.59577	0.022295	-2.56822	3.520735	75.72595
2	KO	forelimb	0.148438	-2.71911	12.29938	-0.44723	7.342847	48.38907
2	KO	forelimb	0.079167	-7.08502	3.140524	-2.61738	6.913192	57.6031
2	KO	forelimb	0.148438	-14.1151	2.999606	-3.67848	4.936537	59.11154
2	KO	forelimb	0.182692	-8.27438	2.323038	-0.62255	6.535151	60.67851
2	KO	forelimb	0.169643	-3.84365	2.29644	-1.20387	5.583294	63.4857
2	KO	forelimb	0.113095	-11.9327	0.398944	-1.05895	8.097848	71.12825
2	KO	forelimb	0.158333	-11.0939	1.767213	-3.3035	2.844087	61.92853
2	KO	forelimb	0.158333	-9.03006	0.135064	-1.46623	9.035758	52.81064
2	KO	forelimb	0.131944	-3.14777	3.92123	-3.09818	2.997148	51.01582
2	KO	forelimb	0.215909	-7.92755	5.191477	-0.90671	6.09249	56.61619
2	KO	forelimb	0.215909	-6.42478	0.574128	-2.91315	6.867731	60.89893
2	KO	forelimb	0.158333	-10.1351	9.19643	-5.99368	8.501738	76.29735
2	KO	forelimb	0.11875	-6.99645	0.92683	-3.93517	3.692412	59.69991
2	KO	forelimb	0.169643	-12.1256	0.635347	-8.99225	0.709171	56.90795
2	KO	forelimb	0.2375	-14.3852	2.072902	-6.41553	6.419146	72.69762
2	KO	forelimb	0.339286	-15.2467	0.887989	-6.33401	6.182963	83.3719
2	KO	forelimb	0.215909	-10.3976	1.599972	-1.04111	9.214787	49.37861
2	KO	forelimb	0.113095	-7.08707	3.361052	-0.79595	4.934002	53.60647
2	KO	forelimb	0.139706	-7.81367	9.72103	-2.58036	9.656628	63.01943
2	KO	forelimb	0.296875	-8.10621	0.985079	-2.97816	5.705161	68.06734
2	KO	forelimb	0.139706	-0.56918	8.133878	-2.22172	7.035735	64.63373
2	KO	forelimb	0.215909	-6.0761	4.068382	-1.10214	6.961104	45.09215
2	KO	forelimb	0.263889	-0.85196	3.401854	-2.16121	7.725047	60.35346

2	KO	forelimb	0.11875	-1.75198	2.189278	-1.5451	4.668172	50.15534
2	KO	forelimb	0.182692	-10.4302	1.248348	-1.44794	8.499738	53.34954
2	KO	forelimb	0.091346	-9.33776	0.430198	-0.96896	8.230217	61.40683
2	KO	forelimb	0.2375	-6.10199	3.149885	-2.87341	11.3275	60.98157
2	KO	forelimb	0.095	-8.25074	2.680553	-3.43413	9.693227	56.16013
2	KO	forelimb	0.339286	-19.1534	1.132995	-2.73507	7.928858	85.10113
2	KO	forelimb	0.2375	-3.94659	0.369805	-0.07072	11.92934	70.6927
2	KO	forelimb	0.215909	-0.65462	7.317825	-3.35444	3.354441	63.09133
2	KO	forelimb	0.148438	-8.31696	2.998482	-3.50609	3.913966	72.64591
2	KO	forelimb	0.11875	-5.81028	0.159155	-0.36077	9.072334	64.36591
2	KO	forelimb	0.131944	-9.4278	0.265966	-1.58768	9.011723	75.71497
2	KO	forelimb	0.169643	-5.97081	2.979414	-4.32571	2.268286	61.17816
2	KO	forelimb	0.158333	-7.33168	4.749982	-0.98838	10.83253	75.62651
2	KO	forelimb	0.11875	-5.30536	1.639271	-4.38563	5.548774	67.76516
2	KO	forelimb	0.139706	-2.3978	5.682661	-1.09107	11.12613	62.21795
2	KO	forelimb	0.263889	-15.6782	2.779974	-1.45377	11.37017	83.67896
2	KO	forelimb	0.158333	-8.07705	0.191625	-3.06966	3.619751	62.35547
2	KO	forelimb	0.113095	-3.22543	1.940036	-2.07634	12.23432	70.86956
2	KO	forelimb	0.076613	-15.7738	5.56214	-3.40393	14.5543	63.96809
2	KO	forelimb	0.131944	-18.5937	0.156203	-0.72487	7.540364	72.33204
2	KO	forelimb	0.11875	-11.3672	2.821882	-6.21163	0.78301	68.07346
2	KO	forelimb	0.098958	-8.72521	8.427761	-0.94996	9.770871	75.7326
2	KO	forelimb	0.158333	-10.8043	2.81889	-2.02685	6.787663	66.42228
2	KO	forelimb	0.11875	-7.11339	9.4472	-1.92321	13.77288	60.13243
2	KO	forelimb	0.148438	-2.9756	2.799144	-1.1488	4.984483	61.04344
2	KO	forelimb	0.113095	-11.2081	1.307089	-2.66756	6.138735	60.50208
2	KO	forelimb	0.131944	-5.19022	9.392763	-3.02655	13.81757	66.40553
2	KO	forelimb	0.131944	-8.65631	5.548694	-1.10534	15.44348	54.16881
2	KO	forelimb	0.148438	-15.9839	2.643896	-8.17379	8.531281	59.03226
2	KO	forelimb	0.59375	-38.2236	2.361292	-0.54559	26.58409	69.7414
2	KO	forelimb	0.296875	-16.467	6.65814	-4.09489	9.040185	68.74696
2	KO	forelimb	0.296875	-17.7568	9.185609	-4.36031	14.17137	77.47938
2	KO	forelimb	0.050532	-9.23834	3.388688	-0.63914	10.79021	54.66698
2	KO	forelimb	0.215909	-2.042	9.043101	-2.71493	6.029128	58.17082
2	WT	hindlimb	0.182692	-0.19251	12.99994	-2.13118	11.8658	54.27938
2	WT	hindlimb	0.087963	-3.75208	6.475598	-2.03125	8.61352	58.53911
2	WT	hindlimb	0.065972	-4.33596	7.464709	-1.88198	8.615837	60.20612
2	WT	hindlimb	0.215909	-0.19338	14.50498	-0.80855	8.385302	55.9771
2	WT	hindlimb	0.263889	-2.15375	9.208656	-1.45983	9.824037	50.85823
2	WT	hindlimb	0.148438	-0.24807	11.58332	-0.19015	8.536214	38.42868
2	WT	hindlimb	0.215909	-2.27538	10.33684	-3.69536	11.34566	54.13137
2	WT	hindlimb	0.296875	-7.4366	15.15835	-4.6407	7.43851	53.21459
2	WT	hindlimb	0.263889	-2.01847	12.34082	-3.87277	16.8995	76.83053
2	WT	hindlimb	0.148438	-2.46463	5.018744	-1.49452	8.600653	58.21188
2	WT	hindlimb	0.169643	-4.01336	8.159113	-0.47018	13.63566	56.67487

2	WT	hindlimb	0.103261	-6.2486	9.572426	-2.15857	15.39131	51.1068
2	WT	hindlimb	0.169643	-6.44532	7.579698	-3.24115	10.47014	52.7508
2	WT	hindlimb	0.131944	-0.46287	10.98968	-4.38628	9.532202	43.34988
2	WT	hindlimb	0.197917	-0.86532	11.03179	-0.81087	14.5484	58.55842
2	WT	hindlimb	0.148438	-1.90407	6.936753	-2.91272	10.58703	52.06938
2	WT	hindlimb	0.158333	-0.36539	16.04032	-0.79877	20.42163	61.89939
2	WT	hindlimb	0.139706	-1.3377	11.99714	-2.77822	15.1863	53.13043
2	WT	hindlimb	0.158333	-2.84855	9.108234	-0.8916	10.37608	48.24831
2	WT	hindlimb	0.2375	-3.6296	8.867494	-1.21698	9.00356	66.90402
2	WT	hindlimb	0.263889	-2.77317	15.7531	-4.14337	12.07622	70.25894
2	WT	hindlimb	0.263889	-3.49987	9.570158	-1.7935	12.60065	65.49602
2	WT	hindlimb	0.215909	-1.82704	12.41619	-1.99759	12.43487	59.42653
2	WT	hindlimb	0.2375	-3.47546	3.315182	-1.1943	13.00012	59.76213
2	WT	hindlimb	0.2375	-1.13408	9.523603	-2.94866	9.46727	55.5175
2	WT	hindlimb	0.11875	-2.90079	14.75302	-0.09701	10.87176	52.20261
2	WT	hindlimb	0.197917	-3.77302	12.73013	-5.20176	15.95619	63.5652
2	WT	hindlimb	0.215909	-1.56494	8.229901	-3.22789	12.66053	62.87298
2	WT	hindlimb	0.296875	-3.74043	14.81152	-6.78507	8.307451	61.61567
2	WT	hindlimb	0.296875	-4.08354	9.406606	-6.16676	16.7906	70.76094
2	WT	hindlimb	0.296875	-6.04008	12.39876	-4.23817	9.383136	46.73794
2	WT	hindlimb	0.113095	-2.27612	13.73383	-1.26674	17.44352	63.86723
2	WT	hindlimb	0.2375	-2.12264	2.961377	-3.58661	14.66121	58.5174
2	WT	hindlimb	0.169643	-8.28521	6.940131	-10.0985	10.01497	58.39742
2	WT	hindlimb	0.263889	-1.96052	4.553393	-0.33528	9.971663	59.86996
2	WT	hindlimb	0.169643	-2.41065	9.065216	-1.15759	13.74072	63.89002
2	WT	hindlimb	0.169643	-1.59306	20.43739	-2.06357	13.46059	66.31201
2	WT	hindlimb	0.2375	-3.2087	8.164331	-7.49674	10.78915	68.33394
2	WT	hindlimb	0.339286	-1.77738	12.72922	-0.99098	8.000153	48.51681
2	WT	hindlimb	0.215909	-2.01459	3.156735	-0.53248	13.56765	60.6659
2	WT	hindlimb	0.148438	-1.48715	12.06024	-0.76901	10.00343	52.77484
2	WT	hindlimb	0.158333	-0.85485	8.610296	-1.14872	13.06578	43.22558
2	WT	hindlimb	0.197917	-1.79875	11.86911	-1.76965	7.111119	65.81617
2	WT	hindlimb	0.197917	-2.21998	4.425998	-2.60143	6.156791	58.95205
2	WT	hindlimb	0.2375	-5.68693	17.58491	-2.33118	13.98786	58.61125
2	WT	hindlimb	0.169643	-7.35696	4.239069	-0.05278	11.3173	56.90134
2	WT	hindlimb	0.215909	-6.17701	6.143037	-0.93132	10.11027	64.56332
2	WT	hindlimb	0.296875	-0.0568	10.84708	-0.48689	8.827809	59.92921
2	WT	hindlimb	0.158333	-2.68262	9.79208	-4.19655	12.94421	57.67257
2	WT	hindlimb	0.182692	-1.14493	12.172	-1.01378	10.97341	40.88774
2	WT	hindlimb	0.197917	-2.93443	0.365544	-0.33931	13.79159	63.66245
2	WT	hindlimb	0.107955	-2.60081	16.03005	-4.47321	6.3323	59.18269
2	WT	hindlimb	0.087963	-3.97817	8.019778	-0.56087	8.092552	44.52526
2	WT	hindlimb	0.215909	-3.98982	8.334361	-2.49385	16.82105	70.19091
2	WT	hindlimb	0.263889	-5.11662	17.44602	-0.0733	7.324978	74.33937
2	WT	hindlimb	0.087963	-4.48442	3.443884	-1.03144	7.451457	41.84086

2	WT	hindlimb	0.197917	-3.88294	9.032835	-1.12921	11.10234	51.81244
2	WT	hindlimb	0.197917	-3.50868	5.680247	-1.93815	8.987493	48.64658
2	WT	hindlimb	0.139706	-0.86903	7.073472	-2.15055	12.83943	50.40292
2	WT	hindlimb	0.074219	-0.73899	6.660025	-3.89015	3.748366	51.20876
2	WT	hindlimb	0.169643	-4.06743	4.577329	-0.98628	18.62751	54.33556
2	WT	hindlimb	0.475	-0.80541	10.79182	-0.60502	4.398139	53.00627
2	WT	hindlimb	0.296875	-0.57045	5.797496	-0.2287	8.631836	53.95267
2	WT	hindlimb	0.339286	-0.41018	15.46796	-0.81415	10.64191	46.31779
2	KO	hindlimb	0.158333	-10.0222	15.14278	-0.57995	10.10057	55.57814
2	KO	hindlimb	0.139706	-4.13077	10.3642	-2.47061	10.9064	63.95435
2	KO	hindlimb	0.125	-2.11446	5.950013	-1.8335	8.22154	46.7458
2	KO	hindlimb	0.113095	-1.89263	5.582881	-0.30494	8.917426	62.32843
2	KO	hindlimb	0.113095	-2.2355	8.147494	-1.67746	11.89821	57.87781
2	KO	hindlimb	0.215909	-3.46064	10.89455	-0.47914	11.02259	55.06973
2	KO	hindlimb	0.296875	-2.63428	12.52506	-4.17744	18.79111	79.67651
2	KO	hindlimb	0.263889	-4.46035	14.46775	-4.01066	10.56088	51.0178
2	KO	hindlimb	0.263889	-3.50671	5.85562	-1.47883	9.940408	61.70117
2	KO	hindlimb	0.296875	-2.67942	11.69263	-2.51815	14.39193	68.89404
2	KO	hindlimb	0.339286	-7.61443	20.14946	-4.38686	14.12383	65.38206
2	KO	hindlimb	0.395833	-1.00913	12.11061	-5.42426	11.89175	66.01806
2	KO	hindlimb	0.339286	-0.63134	6.853164	-0.7331	13.24856	68.22315
2	KO	hindlimb	0.2375	-3.93974	6.848939	-0.90258	11.74083	74.17548
2	KO	hindlimb	0.215909	-0.00887	9.479964	-3.6432	15.4316	72.37264
2	KO	hindlimb	0.197917	-5.45593	9.211747	-5.16354	5.964822	55.29694
2	KO	hindlimb	0.169643	-0.96049	12.31877	-2.85087	9.820937	59.45567
2	KO	hindlimb	0.169643	-3.78157	6.969037	-3.90372	2.691561	53.51164
2	KO	hindlimb	0.11875	-3.54448	9.13951	-4.89752	4.964664	49.8675
2	KO	hindlimb	0.197917	-2.10658	10.7197	-1.41169	11.24512	63.12554
2	KO	hindlimb	0.2375	-6.90314	6.401541	-1.98327	15.555	64.94276
2	KO	hindlimb	0.263889	-0.15986	9.646409	-1.16792	16.82885	73.95724
2	KO	hindlimb	0.148438	-2.63667	6.800585	-1.41393	11.48532	59.81974
2	KO	hindlimb	0.158333	-2.65029	5.337956	-2.81253	6.817487	55.28209
2	KO	hindlimb	0.158333	-3.99697	5.357668	-1.68862	9.410841	52.95388
2	KO	hindlimb	0.169643	-2.94826	2.818976	-1.4698	12.28086	63.27846
2	KO	hindlimb	0.139706	-5.45149	4.21335	-1.07998	12.83317	75.68778
2	KO	hindlimb	0.0625	-2.74374	4.086614	-1.50688	8.3691	64.14988
2	KO	hindlimb	0.215909	-0.71671	14.85978	-0.36167	15.0952	62.39179
2	KO	hindlimb	0.215909	-0.25836	9.099779	-1.28763	13.55194	63.42983
2	KO	hindlimb	0.11875	-0.1114	10.45913	-1.57962	18.63223	78.78628
2	KO	hindlimb	0.158333	-0.86663	12.05296	-4.30966	11.71525	69.9973
2	KO	hindlimb	0.2375	-0.10196	13.15308	-1.57453	15.23335	66.25246
2	KO	hindlimb	0.339286	-0.70703	17.92609	-0.9211	30.88979	79.37462
2	KO	hindlimb	0.131944	-4.60117	2.201968	-0.38176	7.962816	52.46791
2	KO	hindlimb	0.139706	-8.22626	7.824211	-1.58846	13.45197	71.46338
2	KO	hindlimb	0.139706	-6.0647	9.019008	-3.5823	13.32926	72.22046

2	KO	hindlimb	0.296875	-2.51872	12.70821	-0.38692	22.63268	76.2467
2	KO	hindlimb	0.215909	-9.73777	5.351895	-0.90236	12.95963	54.33921
2	KO	hindlimb	0.11875	-7.93305	7.668483	-4.61261	10.6879	61.13925
2	KO	hindlimb	0.197917	-2.09308	14.39824	-0.38626	24.68081	69.22343
2	KO	hindlimb	0.2375	-2.33957	8.951675	-1.51364	14.79083	55.36361
2	KO	hindlimb	0.339286	-0.31226	10.15155	-3.14391	14.59789	63.14954
2	KO	hindlimb	0.182692	-2.05884	8.452141	-1.37386	10.78076	44.53756
2	KO	hindlimb	0.182692	-3.87789	2.807621	-1.5086	17.43155	67.20557
2	KO	hindlimb	0.131944	-2.04663	19.56975	-5.66327	4.282799	54.98287
2	KO	hindlimb	0.169643	-0.6719	9.123858	-1.20748	14.65761	63.30736
2	KO	hindlimb	0.095	-1.08085	6.931534	-1.10099	20.54553	60.59369
2	KO	hindlimb	0.158333	-9.0786	9.238112	-3.25479	11.4743	62.12741
2	KO	hindlimb	0.11875	-1.89609	12.27002	-4.35826	5.563176	58.28753
2	KO	hindlimb	0.139706	-6.60897	17.18633	-0.82541	10.02987	54.61595
2	KO	hindlimb	0.113095	-1.05083	9.684057	-2.71216	10.91172	62.73055
2	KO	hindlimb	0.079167	-1.3311	14.06611	-0.78714	16.43068	51.92645
2	KO	hindlimb	0.069853	-0.269	15.38706	-2.87066	16.05075	55.14163
2	KO	hindlimb	0.158333	-0.19476	13.06702	-1.5669	11.5377	41.79097
2	KO	hindlimb	0.182692	-1.19932	6.627737	-0.15488	17.66717	61.76275
2	KO	hindlimb	0.169643	-6.34021	9.223285	-1.37912	16.57538	57.56407
2	KO	hindlimb	0.158333	-1.43065	11.72892	-1.21789	19.45357	52.60457
2	KO	hindlimb	0.107955	-3.89733	7.593204	-1.94818	21.77204	59.50587
2	KO	hindlimb	0.07197	-5.07029	5.408122	-2.07268	14.35927	59.9153
2	KO	hindlimb	0.296875	-4.15243	12.50762	-3.57881	9.416911	55.83202
2	KO	hindlimb	0.131944	-0.61192	13.75286	-2.74083	16.83011	61.99243
2	KO	hindlimb	0.057927	-2.32322	7.390823	-0.14745	10.15592	47.01882
2	KO	hindlimb	0.11875	-3.22489	12.09373	-0.48967	13.95021	45.97019
2	KO	hindlimb	0.215909	-3.5608	8.388337	-3.97222	11.93115	54.19294
2	KO	hindlimb	0.098958	-3.79647	8.888063	-2.08754	12.38584	41.70185
2	KO	hindlimb	0.139706	-3.07897	17.09263	-4.04665	15.21198	59.15147
4	WT	forelimb	0.475	-29.7536	4.291386	-4.08147	15.87914	63.22953
4	WT	forelimb	0.296875	-27.4891	0.999629	-8.21657	8.216574	80.55386
4	WT	forelimb	0.2375	-26.9439	3.748528	-6.74125	7.167494	65.92462
4	WT	forelimb	0.475	-26.8371	0.353184	-5.0129	0.426145	82.81053
4	KO	forelimb	0.339286	-22.8518	0.512696	-0.90868	4.508547	66.59012
4	KO	forelimb	0.296875	-22.3747	2.887211	-1.4849	7.570611	72.67983
4	KO	forelimb	0.475	-21.1642	9.948603	-1.17124	9.423954	69.98902
4	KO	forelimb	0.395833	-21.051	7.675514	-3.49087	10.98108	76.10263
4	WT	forelimb	0.339286	-20.3138	8.54068	-9.94434	0.207242	69.13003
4	WT	forelimb	0.475	-20.2754	1.414835	-2.04729	2.047294	93.27805
4	WT	forelimb	0.339286	-19.7572	0.585774	-6.71109	1.499438	65.23653
4	WT	forelimb	0.395833	-19.3776	0.533608	-7.65189	12.31054	82.04624
4	KO	forelimb	0.296875	-19.2467	3.982954	-14.202	12.64033	78.33828
4	KO	forelimb	0.296875	-19.0219	3.122062	-4.1692	5.078672	74.3004
4	WT	forelimb	0.296875	-18.4874	0.807771	-13.6372	2.824196	68.42002

4	WT	forelimb	0.2375	-18.333	1.402243	-1.31874	9.407798	70.63718
4	KO	forelimb	0.131944	-18.0016	0.850009	-0.74678	11.043	64.85575
4	KO	forelimb	0.139706	-17.9396	6.008022	-1.29452	11.82232	63.68209
4	KO	forelimb	0.339286	-17.0789	1.82103	-7.7356	7.735595	74.60022
4	WT	forelimb	0.395833	-16.7648	2.065462	-8.67962	3.785421	76.39133
4	WT	forelimb	0.296875	-16.4786	2.052304	-6.79747	2.248757	68.37823
4	WT	forelimb	0.395833	-16.4661	7.785761	-2.32398	5.251109	63.64718
4	WT	forelimb	0.215909	-15.4232	1.432955	-1.73336	9.592387	77.33923
4	WT	forelimb	0.215909	-15.422	2.782133	-1.6336	1.697964	70.5772
4	KO	forelimb	0.148438	-15.419	1.140702	-4.06134	33.83385	60.24166
4	KO	forelimb	0.197917	-14.6463	1.155614	-1.3347	8.699766	59.94635
4	WT	forelimb	0.263889	-14.6016	0.280324	-8.49614	5.196729	71.99393
4	KO	forelimb	0.215909	-14.5606	2.46331	-0.42004	13.65151	69.41161
4	WT	forelimb	0.263889	-14.0974	0.412583	-6.14294	1.690669	68.26822
4	WT	forelimb	0.169643	-13.8845	2.344584	-2.8144	7.034321	62.87919
4	WT	forelimb	0.197917	-13.6831	4.974072	-4.26283	6.905932	67.31914
4	KO	forelimb	0.125	-13.4868	1.799027	-0.80852	10.89566	62.79084
4	WT	forelimb	0.475	-12.9618	2.936421	-3.48523	3.826581	96.43853
4	WT	forelimb	0.475	-12.8471	6.752351	-0.37154	6.430704	72.81191
4	WT	forelimb	0.169643	-12.5805	4.194562	-1.35824	5.252033	50.10142
4	KO	forelimb	0.2375	-12.4732	0.548167	-7.07616	10.17249	85.1954
4	WT	forelimb	0.125	-12.2619	0.354235	-1.93276	6.484838	61.40136
4	KO	forelimb	0.215909	-12.0975	1.067408	-3.34734	2.518099	86.17848
4	WT	forelimb	0.263889	-12.0055	0.722472	-9.87408	2.075056	71.99185
4	WT	forelimb	0.2375	-11.7181	3.326933	-2.4615	5.817768	51.58797
4	WT	forelimb	0.2375	-11.576	0.602593	-1.17492	11.59145	60.4289
4	WT	forelimb	0.395833	-11.4197	2.467486	-10.2073	7.468795	75.07915
4	KO	forelimb	0.131944	-10.9501	0.793329	-0.93593	4.406315	53.78184
4	WT	forelimb	0.107955	-10.7528	1.706948	-2.3147	6.228689	54.9454
4	WT	forelimb	0.197917	-10.7351	5.977424	-3.43228	9.122249	63.32855
4	KO	forelimb	0.2375	-10.4024	0.948736	-0.31813	14.89084	76.2623
4	KO	forelimb	0.11875	-10.3758	0.804549	-0.61791	8.710043	55.94649
4	KO	forelimb	0.091346	-10.2395	0.471446	-0.60541	3.209871	53.53578
4	WT	forelimb	0.2375	-9.95865	1.004576	-1.05664	12.37881	77.99541
4	KO	forelimb	0.197917	-9.83598	3.038543	-4.28294	5.347602	68.1507
4	KO	forelimb	0.2375	-9.63257	2.70962	-7.30775	7.337643	58.69669
4	WT	forelimb	0.395833	-9.39741	6.533034	-3.43726	5.351241	71.17601
4	KO	forelimb	0.263889	-9.29539	3.4631	-4.21677	0.834745	63.90939
4	WT	forelimb	0.125	-9.0823	3.730942	-1.47428	6.974167	59.28629
4	KO	forelimb	0.169643	-8.98255	1.07122	-3.45851	2.038355	74.74904
4	KO	forelimb	0.2375	-8.90301	13.54934	-11.1376	8.203193	64.39906
4	KO	forelimb	0.197917	-8.86066	2.310684	-5.88076	9.457713	54.95614
4	WT	forelimb	0.197917	-8.84926	1.683079	-0.74657	8.085257	59.34334
4	KO	forelimb	0.087963	-8.11252	3.930984	-1.24164	5.867575	69.60771
4	WT	forelimb	0.339286	-8.09497	10.92856	-2.70451	3.878254	63.78847

4	WT	forelimb	0.098958	-7.81083	1.243781	-1.3572	9.006534	45.22921
4	WT	forelimb	0.182692	-7.57223	6.533681	-6.28782	6.287822	63.61123
4	KO	forelimb	0.182692	-7.44594	4.991171	-11.1491	11.14911	75.86163
4	KO	forelimb	0.263889	-7.39829	3.132644	-3.0027	9.430073	69.7624
4	KO	forelimb	0.215909	-7.36049	2.596531	-1.89417	7.337667	65.75567
4	WT	forelimb	0.395833	-7.22438	5.702353	-3.85234	12.54996	68.18407
4	KO	forelimb	0.125	-7.19817	13.70256	-4.1361	0.553132	64.70058
4	WT	forelimb	0.2375	-6.96983	2.114618	-1.17603	7.611386	65.89062
4	KO	forelimb	0.339286	-6.78665	4.321387	-3.52477	6.789226	73.70593
4	KO	forelimb	0.182692	-6.71542	2.53375	-4.92932	1.132402	61.24178
4	KO	forelimb	0.197917	-6.70739	4.997169	-0.13569	9.351507	58.89625
4	KO	forelimb	0.339286	-6.63783	1.609331	-5.51469	6.647527	71.22312
4	KO	forelimb	0.395833	-6.44464	3.213806	-2.90544	5.162524	82.63929
4	KO	forelimb	0.263889	-6.31512	1.018393	-6.60513	6.605131	70.41426
4	WT	forelimb	0.339286	-6.21867	0.034338	-1.65375	8.771288	66.69568
4	WT	forelimb	0.197917	-6.10338	2.825011	-1.32879	13.66056	63.61756
4	WT	forelimb	0.169643	-5.72929	5.492589	-3.17673	3.78243	60.96619
4	KO	forelimb	0.182692	-5.27176	0.714072	-0.43234	8.41888	63.93241
4	WT	forelimb	0.197917	-5.10982	5.395647	-3.88577	9.091321	72.02429
4	KO	forelimb	0.2375	-4.82223	5.258891	-2.01689	12.57765	70.35793
4	KO	forelimb	0.169643	-4.70219	4.232078	-1.23768	20.32656	68.76876
4	WT	forelimb	0.2375	-4.67688	3.574124	-2.72354	5.196344	58.24326
4	WT	forelimb	0.131944	-4.3111	7.491149	-2.6101	7.789149	74.89378
4	WT	forelimb	0.182692	-4.06978	3.783167	-5.89246	6.228494	67.75371
4	WT	forelimb	0.148438	-3.98566	1.481794	-0.10033	10.44158	49.26057
4	WT	forelimb	0.158333	-3.79399	1.908873	-0.8465	11.50621	58.21519
4	WT	forelimb	0.091346	-3.71436	0.999102	-1.86887	2.312005	51.71617
4	WT	forelimb	0.395833	-3.67362	5.577842	-8.78283	3.983408	56.69808
4	KO	forelimb	0.263889	-2.9716	4.355166	-0.87008	14.40418	70.84218
4	KO	forelimb	0.197917	-2.47186	8.464829	-6.37301	3.393237	72.94927
4	KO	forelimb	0.169643	-2.27658	1.91558	-5.1568	1.663462	52.46485
4	KO	forelimb	0.263889	-1.60841	14.17822	-4.83649	6.714416	76.59716
4	WT	forelimb	0.296875	-1.12151	15.04042	-3.06717	18.60416	67.74777
4	KO	forelimb	0.148438	-1.10865	5.641719	-2.96536	5.035162	54.74722
4	WT	forelimb	0.296875	-0.53404	6.619022	-1.75592	6.144716	65.08521
4	KO	forelimb	0.148438	-0.44628	8.138134	-1.26077	7.585929	50.05032
4	WT	forelimb	0.197917	-14.3945	0.53334	-2.15069	8.455897	73.92761
4	KO	forelimb	0.139706	-4.18981	0.587701	-5.94636	1.543013	66.4568
4	WT	forelimb	0.296875	-6.87638	0.614294	-2.75024	5.748861	75.39695
4	WT	forelimb	0.125	-15.8976	0.661738	-0.80856	9.321295	63.30749
4	WT	forelimb	0.395833	-10.7845	1.189371	-0.80891	13.01039	87.6646
4	KO	forelimb	0.081897	-9.88091	1.272141	-6.41061	2.20481	64.96856
4	KO	forelimb	0.169643	-7.7214	1.471644	-3.83972	5.711677	62.89426
4	WT	forelimb	0.2375	-19.5172	1.670217	-1.39376	8.52487	66.15366
4	WT	forelimb	0.339286	-15.1732	1.685104	-11.4466	11.44664	84.21229

4	WT	forelimb	0.182692	-2.7889	1.8063	-0.2344	7.936446	41.10997
4	KO	forelimb	0.475	-17.6553	1.819377	-0.99276	14.41829	83.28666
4	WT	forelimb	0.091346	-6.5661	1.843978	-1.50026	6.181574	55.61347
4	KO	forelimb	0.339286	-15.1276	1.892943	-0.66929	20.19829	95.15836
4	WT	forelimb	0.131944	-11.4528	1.896858	-0.99722	11.84946	64.97618
4	KO	forelimb	0.125	-6.29624	1.921304	-4.45892	3.354188	59.50633
4	KO	forelimb	0.395833	-27.0216	1.988843	-1.12211	25.33193	76.7383
4	WT	forelimb	0.339286	-4.88844	2.334797	-1.97788	10.00038	57.61132
4	KO	forelimb	0.139706	-11.2322	2.337006	-2.9024	9.964386	61.67678
4	WT	forelimb	0.2375	-6.33147	2.460184	-5.19261	0.593898	58.97931
4	WT	forelimb	0.296875	-19.1352	3.011915	-1.59961	6.822087	73.6367
4	KO	forelimb	0.475	-11.9795	3.103992	-4.20069	6.676628	81.27905
4	WT	forelimb	0.079167	-10.7902	3.155959	-1.36035	9.805712	63.41157
4	KO	forelimb	0.095	-2.60668	3.832473	-5.61855	2.529573	61.61008
4	KO	forelimb	0.296875	-4.05481	4.054808	-6.4451	10.44022	75.04716
4	WT	forelimb	0.148438	-3.94824	4.886743	-2.66849	5.896865	55.64237
4	KO	forelimb	0.148438	-13.5213	5.368034	-9.32403	46.30649	57.0302
4	KO	forelimb	0.158333	-3.88937	6.094642	-6.05203	10.6609	70.37628
4	KO	forelimb	0.475	-16.1014	6.688265	-4.70469	18.79977	96.83725
4	KO	forelimb	0.084821	-7.34728	6.817954	-0.11143	5.917613	54.43187
4	WT	hindlimb	0.2375	-11.965	9.577343	-0.20717	9.429331	46.86327
4	WT	hindlimb	0.339286	-11.8855	14.21468	-7.57807	10.6451	49.703
4	WT	hindlimb	0.475	-11.1816	7.176834	-4.31854	12.26979	56.88149
4	WT	hindlimb	0.395833	-9.62988	4.510072	-0.98073	12.28168	47.07312
4	KO	hindlimb	0.11875	-9.62383	4.415865	-0.30267	13.7801	82.12256
4	KO	hindlimb	0.182692	-9.31666	5.487555	-2.98782	12.92128	81.1307
4	KO	hindlimb	0.475	-9.09234	10.95978	-0.96007	17.31926	86.84795
4	WT	hindlimb	0.169643	-7.76889	6.830678	-4.25941	4.848673	56.13843
4	WT	hindlimb	0.263889	-7.66992	9.546166	-2.33076	9.615101	56.97898
4	KO	hindlimb	0.475	-7.57715	27.04812	-2.25555	38.04949	79.97283
4	WT	hindlimb	0.107955	-7.55899	13.47374	-6.64881	25.49292	62.19949
4	KO	hindlimb	0.182692	-7.51368	6.820321	-1.14199	7.267323	70.62548
4	KO	hindlimb	0.395833	-7.42418	3.220694	-0.7155	14.5288	68.20157
4	WT	hindlimb	0.148438	-6.84853	11.06086	-4.29596	10.1214	57.0021
4	WT	hindlimb	0.139706	-6.83719	8.960681	-0.43893	14.01335	60.03215
4	WT	hindlimb	0.182692	-6.71856	6.117445	-0.44681	17.78965	67.68165
4	WT	hindlimb	0.197917	-6.46986	5.271799	-1.39293	37.5602	81.32647
4	WT	hindlimb	0.158333	-6.31939	3.322176	-3.82872	5.583408	53.53479
4	KO	hindlimb	0.158333	-6.29529	4.931943	-0.69434	16.55541	65.49611
4	WT	hindlimb	0.475	-6.23382	4.269567	-3.3571	12.60576	62.87652
4	KO	hindlimb	0.263889	-6.09727	5.702043	-5.9057	21.59907	66.26572
4	WT	hindlimb	0.215909	-6.02608	9.77385	-1.35004	7.602619	38.60942
4	KO	hindlimb	0.263889	-5.49101	6.55893	-1.56633	15.45616	63.91127
4	KO	hindlimb	0.081897	-5.46121	6.099425	-0.97262	8.36751	55.69364
4	WT	hindlimb	0.169643	-5.2351	8.591301	-1.49176	13.00731	57.63814

4	KO	hindlimb	0.339286	-5.22237	5.200825	-8.40883	13.58786	65.80963
4	KO	hindlimb	0.148438	-5.17084	11.6383	-1.13195	11.61763	46.46457
4	KO	hindlimb	0.158333	-4.96417	13.67066	-2.97641	9.791934	45.68072
4	KO	hindlimb	0.339286	-4.94157	7.633113	-0.67907	14.36493	63.43314
4	KO	hindlimb	0.475	-4.86411	4.949213	-0.18327	18.98018	65.37414
4	WT	hindlimb	0.395833	-4.84303	11.51153	-3.35562	16.72005	70.33974
4	WT	hindlimb	0.215909	-4.51755	13.05955	-1.24782	10.00731	44.49399
4	WT	hindlimb	0.182692	-4.26163	6.484966	-6.45839	11.90619	51.44466
4	KO	hindlimb	0.095	-4.25271	9.403899	-0.53756	11.91723	49.70753
4	WT	hindlimb	0.215909	-4.23287	5.180073	-0.36986	18.79178	72.57624
4	KO	hindlimb	0.113095	-4.20772	7.650928	-0.27549	19.50103	52.06584
4	WT	hindlimb	0.148438	-4.1286	7.559395	-2.40461	9.149937	49.93501
4	WT	hindlimb	0.2375	-3.74272	8.583198	-1.08705	23.48304	61.15435
4	WT	hindlimb	0.296875	-3.62843	1.504707	-3.91332	21.39883	69.52625
4	KO	hindlimb	0.081897	-3.52806	5.869773	-1.34115	10.40367	65.51118
4	KO	hindlimb	0.139706	-3.49998	6.680242	-0.41338	17.69306	58.36054
4	KO	hindlimb	0.197917	-3.43936	18.31247	-2.67944	19.96506	66.0371
4	KO	hindlimb	0.148438	-3.39645	2.162272	-0.68186	10.20891	43.01672
4	KO	hindlimb	0.139706	-3.34664	6.681575	-3.13774	7.419696	66.08544
4	KO	hindlimb	0.158333	-3.19162	11.95922	-0.13394	19.50106	58.38522
4	WT	hindlimb	0.296875	-3.03142	3.353443	-1.20297	6.914949	43.6367
4	WT	hindlimb	0.158333	-3.01032	5.091969	-5.1872	1.905082	49.64357
4	KO	hindlimb	0.182692	-3.00667	11.67965	-11.5355	9.860493	62.89799
4	KO	hindlimb	0.125	-2.87851	8.692079	-1.98066	8.62299	63.81305
4	KO	hindlimb	0.139706	-2.82732	11.44024	-1.66505	11.74563	62.83939
4	WT	hindlimb	0.395833	-2.77383	3.947375	-3.64514	8.736867	55.39015
4	WT	hindlimb	0.339286	-2.55048	17.0902	-6.43743	17.85269	67.9512
4	KO	hindlimb	0.2375	-2.32157	7.155088	-2.82511	19.02461	69.29847
4	KO	hindlimb	0.139706	-2.2543	3.378802	-4.32929	8.532084	56.55086
4	WT	hindlimb	0.2375	-2.1541	7.813743	-1.08707	17.06389	49.08418
4	KO	hindlimb	0.087963	-2.13703	6.169721	-1.9799	10.84006	62.37189
4	WT	hindlimb	0.395833	-2.02585	9.135494	-1.36568	10.75182	62.29363
4	WT	hindlimb	0.158333	-1.76663	12.21067	-1.47133	19.53597	41.24782
4	KO	hindlimb	0.339286	-1.73354	9.191798	-3.71055	2.785167	66.51233
4	KO	hindlimb	0.087963	-1.7334	13.87647	-1.80702	9.102886	62.55434
4	KO	hindlimb	0.296875	-1.54711	11.22888	-4.92911	0.322656	66.88858
4	KO	hindlimb	0.215909	-1.44757	3.95631	-4.72708	12.20664	82.91127
4	WT	hindlimb	0.148438	-1.4403	8.45921	-3.14206	14.30265	52.64493
4	KO	hindlimb	0.475	-1.3709	14.41532	-5.75224	19.87683	78.67653
4	KO	hindlimb	0.113095	-1.33291	25.08447	-3.682	12.29025	68.52106
4	KO	hindlimb	0.095	-1.26124	7.771988	-0.44155	9.886007	48.48106
4	KO	hindlimb	0.395833	-1.1666	4.06976	-1.97298	8.523458	75.28747
4	KO	hindlimb	0.339286	-1.11461	8.071787	-3.34839	11.7518	67.4231
4	WT	hindlimb	0.169643	-1.0448	8.932008	-0.49181	11.41117	45.36018
4	WT	hindlimb	0.113095	-1.00965	8.694277	-0.13851	9.280219	46.74798

4	WT	hindlimb	0.197917	-1.00793	4.747917	-2.9679	16.48098	59.50928
4	WT	hindlimb	0.339286	-0.9872	9.833537	-9.37968	1.806177	56.50109
4	WT	hindlimb	0.169643	-0.91159	10.93082	-3.10178	24.33723	68.44566
4	WT	hindlimb	0.11875	-0.75452	6.627156	-4.17748	7.00028	49.23171
4	WT	hindlimb	0.215909	-0.66103	9.127129	-1.2911	19.14752	48.58439
4	KO	hindlimb	0.2375	-0.59303	7.149274	-2.57383	21.80033	80.34953
4	KO	hindlimb	0.148438	-0.57319	2.587057	-0.5894	8.004827	51.83816
4	KO	hindlimb	0.2375	-0.53167	8.346281	-3.68584	12.74705	64.11309
4	KO	hindlimb	0.169643	-0.34441	14.95648	-8.01697	1.666336	55.59598
4	WT	hindlimb	0.103261	-0.29009	12.08219	-2.00338	8.282002	46.21337
4	WT	hindlimb	0.2375	-0.10682	9.479259	-0.97589	15.69305	63.47779
4	WT	hindlimb	0.296875	-3.63912	2.685628	-0.96067	25.37813	79.15773
4	WT	hindlimb	0.339286	-9.93057	4.254042	-3.60919	19.21406	80.18794
4	WT	hindlimb	0.197917	-5.39214	4.555799	-2.41053	12.41377	61.96453
4	KO	hindlimb	0.296875	-4.68746	5.245122	-11.5617	12.71782	65.20174
4	KO	hindlimb	0.084821	-4.36779	5.368943	-2.49061	15.81364	57.96845
4	KO	hindlimb	0.197917	-1.16726	5.893788	-4.36638	12.37262	69.45014
4	WT	hindlimb	0.107955	-1.3929	5.928104	-2.02028	9.438028	52.64126
4	KO	hindlimb	0.087963	-2.91741	6.088683	-0.25662	15.11561	60.25729
4	WT	hindlimb	0.139706	-3.51078	6.42712	-0.24848	8.162272	37.30974
4	KO	hindlimb	0.148438	-2.85541	6.578166	-4.35054	16.75416	69.22906
4	KO	hindlimb	0.182692	-1.12823	7.042794	-7.38092	7.380916	52.04787
4	KO	hindlimb	0.215909	-4.38522	9.716965	-0.95015	7.916867	62.50565
4	KO	hindlimb	0.131944	-0.54803	10.39598	-0.33656	8.632465	61.18374
4	WT	hindlimb	0.215909	-3.28173	10.56941	-0.19893	27.81543	60.08521
4	WT	hindlimb	0.197917	-2.90295	12.09527	-0.59677	13.61624	54.28929
